# Molecular Subtypes of Oral Squamous Cell Carcinoma Based on Immunosuppression Genes Using a Deep Learning Approach

**DOI:** 10.3389/fcell.2021.687245

**Published:** 2021-08-05

**Authors:** Simin Li, Zhaoyi Mai, Wenli Gu, Anthony Chukwunonso Ogbuehi, Aneesha Acharya, George Pelekos, Wanchen Ning, Xiangqiong Liu, Yupei Deng, Hanluo Li, Bernd Lethaus, Vuk Savkovic, Rüdiger Zimmerer, Dirk Ziebolz, Gerhard Schmalz, Hao Wang, Hui Xiao, Jianjiang Zhao

**Affiliations:** ^1^Stomatological Hospital, Southern Medical University, Guangzhou, China; ^2^Faculty of Physics, University of Münster (Westfälische Wilhelms-Universität Münster), Münster, Germany; ^3^Dr. D. Y. Patil Dental College and Hospital, Dr. D. Y. Patil Vidyapeeth, Pune, India; ^4^Faculty of Dentistry, University of Hong Kong, Hong Kong, China; ^5^Laboratory of Molecular Cell Biology, Beijing Tibetan Hospital, China Tibetology Research Center, Beijing, China; ^6^Department of Cranio Maxillofacial Surgery, University Clinic Leipzig, Leipzig, Germany; ^7^Department of Cariology, Endodontology and Periodontology, University of Leipzig, Leipzig, Germany; ^8^Shanghai Tenth People’s Hospital, Tongji University, Shanghai, China; ^9^Shenzhen Stomatological Hospital, Southern Medical University, Shenzhen, China

**Keywords:** immunosuppression, oral squamous cell carcinoma, survival, deep learning, bioinformatics

## Abstract

**Background:** The mechanisms through which immunosuppressed patients bear increased risk and worse survival in oral squamous cell carcinoma (OSCC) are unclear. Here, we used deep learning to investigate the genetic mechanisms underlying immunosuppression in the survival of OSCC patients, especially from the aspect of various survival-related subtypes.

**Materials and methods:** OSCC samples data were obtained from The Cancer Genome Atlas (TCGA), International Cancer Genome Consortium (ICGC), and OSCC-related genetic datasets with survival data in the National Center for Biotechnology Information (NCBI). Immunosuppression genes (ISGs) were obtained from the HisgAtlas and DisGeNET databases. Survival analyses were performed to identify the ISGs with significant prognostic values in OSCC. A deep learning (DL)-based model was established for robustly differentiating the survival subpopulations of OSCC samples. In order to understand the characteristics of the different survival-risk subtypes of OSCC samples, differential expression analysis and functional enrichment analysis were performed.

**Results:** A total of 317 OSCC samples were divided into one inferring cohort (TCGA) and four confirmation cohorts (ICGC set, GSE41613, GSE42743, and GSE75538). Eleven ISGs (i.e., BGLAP, CALCA, CTLA4, CXCL8, FGFR3, HPRT1, IL22, ORMDL3, TLR3, SPHK1, and INHBB) showed prognostic value in OSCC. The DL-based model provided two optimal subgroups of TCGA-OSCC samples with significant differences (*p* = 4.91E-22) and good model fitness [concordance index (C-index) = 0.77]. The DL model was validated by using four external confirmation cohorts: ICGC cohort (*n* = 40, C-index = 0.39), GSE41613 dataset (*n* = 97, C-index = 0.86), GSE42743 dataset (*n* = 71, C-index = 0.87), and GSE75538 dataset (*n* = 14, C-index = 0.48). Importantly, subtype Sub1 demonstrated a lower probability of survival and thus a more aggressive nature compared with subtype Sub2. ISGs in subtype Sub1 were enriched in the tumor-infiltrating immune cells-related pathways and cancer progression-related pathways, while those in subtype Sub2 were enriched in the metabolism-related pathways.

**Conclusion:** The two survival subtypes of OSCC identified by deep learning can benefit clinical practitioners to divide immunocompromised patients with oral cancer into two subpopulations and give them target drugs and thus might be helpful for improving the survival of these patients and providing novel therapeutic strategies in the precision medicine area.

## Introduction

Tumor cells can produce a variety of immunosuppressive factors that can inhibit the normal antitumor functions of immune cells, such as tumor-associated macrophages (TAMs), tumor-associated neutrophils (TANs), cancer-associated fibroblasts (CAFs), and regulatory T cells (Tregs; Liu and Cao, 2016). By the immunosuppression mechanisms mediated by the interaction between tumor cells and immune cells, tumor cells can escape elimination from immune surveillance and tumor immunity, thereby further contributing to cancer progression (Kim et al., 2007). Immunosuppression is involved in oral squamous cell carcinoma (OSCC) pathogenesis and contributes to its increased incidence and poor cancer-specific outcomes (Chang et al., 2020). Patients with a history of immunosuppression (e.g., organ transplant, autoimmune disease, pulmonary disorder, hematological malignancy, myeloproliferative disorder, and HIV infection) have been shown to have an increased risk of a second malignancy in OSCC (Tota et al., 2018). In addition, immunosuppression is significantly associated with poor outcomes of OSCC, and immunosuppressed patients have been shown to have an approximately twofold increase in the cancer-specific outcomes (e.g., recurrence and overall survival) compared with non-immunosuppressed individuals (Margalit et al., 2016).

The application of immunosuppressive drug agents (e.g., calcineurin inhibitors, antiproliferative agents, mTOR inhibitor, and steroids) can inhibit the strength and activity of the immune system by affecting the expression of many immunosuppression genes (ISGs) {e.g., cytokines [interleukin (IL)-2, IL-4, IL-6, IL-15, IL-18, IL-23, interferon gamma (IFN-γ), and tumor necrosis factor alpha (TNF-α)] and chemoattractant chemokines (e.g., CCL-2, CXCL-9, and CXCL-10)} (Misra et al., 2020). Although the dysregulation of ISGs has been shown to contribute to the carcinogenesis of oral cancers (Jewett et al., 2006), the specific ISGs and their mediated signaling pathways involved in the pathogenesis of OSCC have not yet been identified from a comprehensive and systematic aspect. The identification of ISGs as biomarkers in OSCC might have significant value for the clinical practice: on the one hand, selected ISGs could be used for evaluating the incidence risk and prognosis of OSCC; on the other hand, these could be regarded as therapeutic targets for improved OSCC management.

In order to address this research gap, bioinformatic analyses were performed based on the human ISGs obtained from HisgAtlas (Liu et al., 2017) and DisGeNET (Piñero et al., 2017), and the immunosuppressive drug agents were downloaded from the DrugBank (Wishart et al., 2018). Gene expression data regarding OSCC was collected from the TCGA databases (Tomczak et al., 2015). Deep learning (DL), a machine learning method harvesting Artificial Intelligence, has shown high impact in cancer research for classifying the subtypes of cancer samples in liver cancer (Chaudhary et al., 2018), breast cancer (Rohani and Eslahchi, 2020), lung cancer (Asuntha and Srinivasan, 2020), and head and neck cancer (Zhao Z. et al., 2020). Since genetic heterogeneity is a common feature of different tumors, molecular subtyping of cancers could be very helpful in devising precision medicine approaches for treating each subtype of cancer patients (Koo et al., 2016). Therefore, a DL-based model was applied in this research for classifying OSCC patients into molecular subtypes based on their significant feature ISGs’ biological functions.

Thus, this study aimed to investigate the genetic mechanisms of immunosuppression in the pathogenesis of OSCC using bioinformatics analyses to identify the ISGs with significant prognostic value in OSCC, and the ISGs-involving pathways enriched in the aggressive subtype of OSCC, as differentiated by a deep learning model.

## Materials and Methods

### The Study Design

**FIGURE 1:**
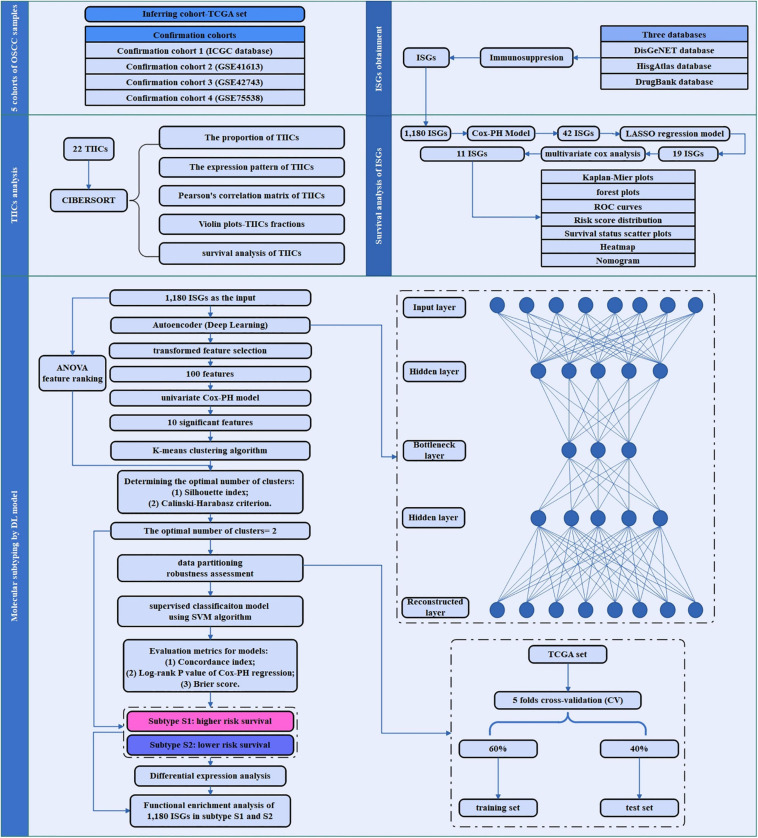
The study flowchart of the current research. This flowchart consisted of five sections: sample data collection of five cohorts of OSCC sample; ISGs obtainment, TIICs analysis, survival analysis of ISGs, and molecular subtyping by DL-based model.

An overview of the workflow of this study is depicted in Figure 1. In brief, the TCGA set as inferring set and four confirmation cohorts were downloaded, and the data information in these datasets is shown in Table 1. The ISGs were obtained from three databases [DisGeNET (Piñero et al., 2017), HisgAtlas (Liu et al., 2017), and DrugBank (Wishart et al., 2018)]. First, the proportion and expression profiling of 22 TIICs were analyzed by using CIBERSORT. Second, the survival analysis was performed to screen out the ISGs that were most significantly related to prognosis. Afterward, the deep learning-based model was constructed to achieve the compression and transformation of gene features. These reduced new gene features were used for clustering the samples by the K-means clustering algorithm. Then, a supervised classification model was constructed by using the support vector machine (SVM) algorithm. In order to investigate the difference between the subtypes identified by the deep learning-based model, the functional enrichment analysis was performed to identify the functional difference of the ISGs enriched in varying subtypes.

**TABLE 1:** The detailed data information (i.e., genes number, sample size in the expression profile, sample size with the clinical information, and events of dead and alive, respectively) of the inferring (TCGA dataset) and cohort datasets (i.e., GSE41613, GSE42743, GSE75538, and ICGC dataset) analyzed in the current research.

Dataset	Genes number	Samples from the expression profile	Samples with clinical information	Events (alive)	Events (dead)
TCGA	19754	335	317	203	114
GSE41613	23516	97	97	46	51
GSE42743	23516	103	71	31	40
GSE75538	20818	28	14	11	3
ICGC	24003	40	40	32	8

### Data Procurement

#### Inferring Cohort

Head and neck squamous cell carcinoma (HNSCC) data were obtained from the TCGA portal^1^ (Tomczak et al., 2015). Based on the TCGA-Assembler 2 (Version 2.0.6^2^) (Wei et al., 2018), the HNSCC samples with the RNA sequencing (RNA-Seq) data (UNC IlluminaHiSeq_RNASeqV2; Level 3) and the clinical information were obtained. Among the HNSCC data, a total of 317 OSCC samples were selected by choosing the specific anatomic sites, including buccal mucosa, alveolar ridge, floor of mouth, hard palate, oral cavity, and the anterior two-thirds of the tongue (Krishna et al., 2014). In order to normalize and preprocess the data, three steps introduced by Wang et al. were performed to deal with the missing values (Wang et al., 2014). First, the biological features [e.g., genes/microRNAs (miRNAs)] were removed if they have zero value in more than 20% of patients. The samples were removed if they have missing values across more than 20% features. Second, we used the impute function from the R impute package (Xiang et al., 2008) to fill out the missing values. Third, we removed input features with zero values across all samples.

#### Confirmation Cohorts

##### Confirmation cohort 1 (ICGC cohort and RNA-Seq)

A total of 178 OSCC samples with RNA-Seq data were obtained from the International Cancer Genome Consortium (ICGC) portal^3^ (Zhang et al., 2011). Among these 178 OSCC samples, 40 samples with the survival information were selected. This cohort was used for validating the analyzing results.

##### Confirmation cohort 2 (NCI cohort, microarray gene expression, and GSE41613)

A total of 97 samples with survival information were chosen from the GSE41613 microarray dataset^4^ (, which was from a study of patients with OSCC (Lohavanichbutr et al., 2013). The experimental platform of this dataset is based on GPL570 [HG-U133_Plus_2] Affymetrix Human Genome U133 Plus 2.0 Array.

##### Confirmation cohort 3 (NCI cohort, microarray gene expression, and GSE42743)

A total of 97 samples with survival information were chosen from the GSE42743 microarray dataset^5^ (, which was from a study of patients with OSCC (Lohavanichbutr et al., 2013). The experimental platform of this dataset is based on GPL570 [HG-U133_Plus_2] Affymetrix Human Genome U133 Plus 2.0 Array.

##### Confirmation cohort 4 (NCI cohort, microarray gene expression, and GSE75538)

A total of 14 samples with survival information were chosen from the GSE75538 microarray dataset^6^ (, which was from a study of patients with OSCC (Krishnan et al., 2016). The experimental platform of this dataset is based on GPL18281 Illumina Human HT-12 WG-DASL V4.0 R2 expression beadchip.

### Procurement of ISGs

The immunosuppression-related genes were downloaded from DisGeNET database^7^ (Piñero et al., 2017), HisgAtlas database^8^ (Liu et al., 2017), and Drugbank database^9^ (Wishart et al., 2018). After combining ISGs obtained from the above three databases, a total of 1,181 immunosuppressant genes were obtained. Afterward, the expression profiling of these 1,181 immunosuppressant genes was extracted from the OSCC datasets. Among these 1,181 ISGs, the expression level of the only one gene TNFRSF6B in the OSCC samples was zero and thus removed, and finally, 1,180 ISGs were used for the subsequent analysis.

### Analysis of Tumor-Infiltrating Immune Cells in OSCC Samples

Twenty-two tumor-infiltrating immune cells (TIICs) were obtained based on the CIBERSORT webtool^10^ (Chen et al., 2018). First, the expression profiles of ISGs in the OSCC samples were normalized, and the proportion of tumor-infiltrating immune cells (TIICs) in OSCC and healthy control samples were predicted by the CIBERSORT webtool. Second, the expression levels of varying ISGs in each type of cell were obtained. The average value of all ISGs in a certain type of cell was regarded as the expression levels of this type of cell in samples. The heatmap was plotted to show the expression levels of 22 TIICs in 151 samples (3 healthy samples and 148 OSCC samples). Third, the correlation plot was drawn based on the expression levels of TIICs in 151 samples in order to analyze the correlation between TIICs in the pathogenesis of OSCC. The Pearson correlation coefficient was used for calculating the correlation between any two types of TIICs. In addition, the Wilcox test was used for examining the differential expression status of each TIIC in OSCC samples compared with the healthy samples. Afterward, Kaplan–Meier analysis was utilized to investigate the prognostic value of 22 tumors infiltrating immune cells in OSCC tissues.

### The Survival Analysis of ISGs

The OSCC patients were divided into two groups (i.e., high expression group and low expression group) according to the median value of gene expression levels of ISGs. The R package survival (Lin and Zelterman, 2002) was employed to mine ISGs that were significant prognostic indicators using Cox proportional hazards model. The ISGs with a significance level < 0.01 were selected to be survival-associated ISGs. Regarding these survival-related ISGs, least absolute shrinkage and selection operator (LASSO) regression analysis was performed to further screen the genes that were more representative of prognosis. As for the ISGs obtained by LASSO regression analysis, multivariate Cox regression analysis was performed, and Akaike information criterion (AIC) was used for optimizing the statistical model, and the ISGs that were the most representative of prognosis were finally identified and defined as “risk ISGs.” Afterward, a series of analyses were performed on the risk ISGs. First, the hazard ratio (HR) and 95% confidence interval (CI) were calculated from the univariate Cox proportional hazards regression model. Cox regression coefficients are directly related to hazard rates, where positive coefficients represent unfavorable prognosis (HR > 0.1) and negative coefficients exert protective effects (HR < 0.1). Based on the HR and CI values, the forest plot for the risk ISGs of the multivariable model was plotted.

Afterward, Kaplan–Meier survival analysis was performed to investigate the prognostic value of the risk ISGs in OSCC; Receiver operating characteristic (ROC) curve analysis by “survivalROC” package in R program was performed to assess the predictive accuracy of these risk ISGs’ prognostic value for time-dependent cancer death. In the next step, risk curves analyses were performed to show the relationship between the survival status of patients and the risk score of genes. Multivariate Cox proportional hazards regression model was used to calculate the risk score based on the risk ISGs and the impact of OS information. The risk score of each sample was calculated using the formula of risk score = β1Exp1 + β2Exp2 + … + βxExpx (βi, the coefficient value; Expx, the gene expression level). The OSCC patients were classified into low- and high-risk groups according to the median RS survival analysis, and log-rank test was performed to evaluate the differences between the two groups. Furthermore, the nomogram was plotted to show the relationship between the expression levels of risk ISGs and survival time of OSCC patients. Afterward, the four clinical features (i.e., age, gender, risk score, and pathological stage) of OSCC samples were extracted from the TCGA database, ICGC database, and three datasets (i.e., GSE41613, GSE42743, and GSE75538), and thus, the nomogram related to the clinical features was plotted.

### The Molecular Subtyping of OSCC Samples

The methods of this section for molecular subtyping of cancer samples mainly followed the methods described in detail by Chaudhary et al. (2017; 12). Briefly, an autoencoder with three hidden layers (500, 100, and 500 nodes, respectively) was implemented, and DL framework was constructed. The initial number (1,180) of ISGs gene features was compressed to 100 new gene features. For each of these transformed new gene features generated by the autoencoder, we built a univariate Cox proportional hazards (Cox-PH) model and selected features from which a significant Cox-PH model was obtained (log-rank *p* < 0.05). These 100 new gene features were used to cluster OSCC samples using the K-means clustering algorithm. The two metrics (Silhouette index and Calinski–Harabasz criterion) were used for determining the optimal number of clusters. The cross-validation (CV)-like procedure was used for data partition of TCGA data: 60% (training set) and 40% (test set). The supervised classification model using SVM algorithm was constructed. Afterward, three sets of evaluation metrics (i.e., Concordance index, log-rank *p*-value of Cox-PH regression, and Brier score) were used for evaluating the accuracy of survival prediction in the subgroups identified by the above-described methods. In addition, the performances of the DL framework were compared with an alternative approach—principal component analysis (PCA).

### The Difference Between Subtypes of OSCC Samples

Based on the subtypes obtained by deep learning algorithm and K-means clustering, the differential expression analysis was performed by using the DESeq2 package (version 1.36.0) (Love et al., 2014) to identify the differentially expressed genes (DEGs) between the varying subtypes (*p* < 0.05, and llogFCl > 1). The heatmap was plotted to show the expression profiling of DEGs expressed in varying subtypes of OSCC. Most importantly, the Gene Set Enrichment Analysis (GSEA) was performed to identify the Kyoto Encyclopedia of Genes and Genomes (KEGG) signaling pathways of 1,180 ISGs in the varying subtypes, respectively. By performing the functional enrichment analysis, the difference between the function of varying subtypes can be identified. The significantly enriched signaling pathways with *p* < 0.05 were selected for the varying subtypes, respectively, and thus, the ISGs pathways for the varying subtypes were constructed by using Cytoscape (version 3.6.1) (Shannon et al., 2003).

Afterward, the protein–protein interaction pairs of 1,180 ISGs were obtained from the Human Protein Reference Database (HPRD; Keshava Prasad et al., 2009), and thus, an ISGs-related PPI network was constructed by using Cytoscape software (version 3.6.1) (Shannon et al., 2003). The DEGs dysregulated between these two subtypes were mapped to this PPI network. The topological characteristics of nodes in this PPI network were analyzed. In addition, the drugs targeting the ISGs were downloaded from the DrugBank database, and thus, an ISGs–target drug interaction network was constructed by using Cytoscape software (version 3.6.1) (Shannon et al., 2003). The DEGs dysregulated between subtypes were mapped to this network.

## Results

### Tumor-Infiltrating Immune Cells in OSCC Samples

**FIGURE 2:**
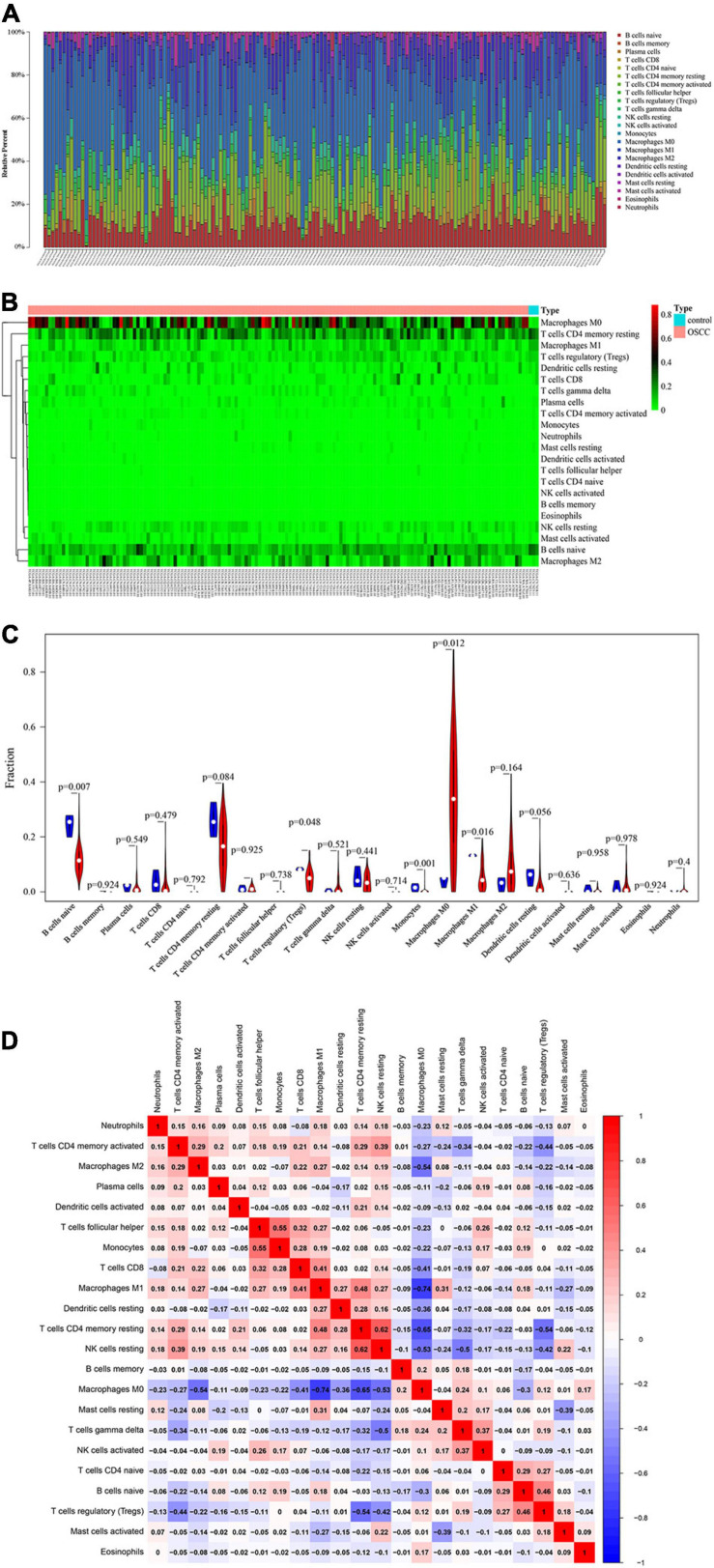
Performance of CIBERORT across TIICs in OSCC. **(A)** The distribution of 22 tumor-infiltrating immune cells (TIICs) in OSCC samples. The abscissa represents the name of 151 samples, and the ordinate represents the composition ratio of the cells in each sample. Different color represents different types of cells. The longer column of each cell in a certain sample indicates that the proportion of this type of cell is higher in this sample. **(B)** Heat map of the 22 TIICs proportions in 148 OSCC and 3 healthy control samples. Each column represents a sample, and each row represents one type of immune cell population. The levels of the immune cell populations are shown in different colors, which transition from green to red with increasing proportions. The abscissa represents the 151 samples: The pink represents the 148 OSCC samples, and the sky blue represents the 3 healthy samples. The ordinate represents the expression levels of TIICs in each sample. In the color bar, green represents the low expression of TIICs in samples, red represents the high expression of TIICs in samples, and black represents that the TIICs were not expressed in the samples, meaning the expression level was zero. **(C)** The differential expression status of TIICs in 151 samples. Violin plot of OSCC samples and adjacent healthy samples groups for the target cohort. Red means the OSCC samples group, and blue represents the adjacent healthy samples group; white dots indicate the average expression level of TIICs in all samples. The *p*-value represents the differential expression status of TIICs in OSCC samples compared with healthy samples. The abscissa represents the TIICs, and the ordinate represents the overall expression status of each TIIC in all the 151 samples. **(D)** Correlation matrix of 22 immune cell proportions and immune/stromal score in OSCC. Variables have been ordered by average linkage clustering. For comparison, immune/stromal score has been rescaled to range between zero and one separately in each study. The correlation between TIICs in the pathogenesis of OSCC. Both the abscissa and ordinate represent the 22 types of TIICs. The color bar shows the correlation value of TIICs. Blue means the TIICs were negatively correlated, and red means the TIICs were positively correlated. The darker color means the correlation was more significant. The diagonal line drawn from coordinate (0,22) to coordinate (22,0) has a correlation of 1.

A total of 151 samples, including 148 OSCC samples and 3 healthy control samples, were obtained by selecting samples with a *p* < 0.05 from the CIBERSORT webtool. The proportion of 22 TIICs in each sample is shown in Figure 2A. The distribution proportion of macrophages M0, M1, and M2 was obviously shown to be the greatest in most of all samples, indicating that macrophages are playing more critical roles in the development of OSCC than the other types of TIICs.

The heatmap shows the expression levels of 22 TIICs in 151 samples (Figure 2B). As clearly observed from Figure 2B, macrophages M0 were highly expressed in the OSCC samples, and the other types of cells were downregulated or nearly non-expressed in the OSCC samples. The differential expression of 22 TIICs in 151 samples is shown in Figure 2C. In terms of macrophages (i.e., M0, M1, and M2), macrophages M0 occupying the highest fraction (approximately 90%) were significantly highly expressed in OSCC samples compared to the healthy control samples (*p* = 0.012), and macrophages M1 occupying approximately 20% among all immune cells were significantly lowly expressed in OSCC samples compared to the healthy control samples (*p* = 0.016), while macrophage M2 occupying approximately 40% among all immune cells did not show significant expression changes between OSCC samples and healthy control samples (*p* = 0.164 > 0.05). As for the other TIICs except macrophages, naive B cells and regulatory T cells (Treg) were found to be lowly expressed in OSCC samples compared to the healthy control samples [*p* = 0.007 (naive B cells) and p (0.048 (Treg)]. In addition, there were no statistical differences in expression levels between OSCC and healthy control samples and as for memory B cells (p = 0.924), plasma cells (*p* = 0.549), CD8 T cells (*p* = 0.479), naive CD4 T cells (*p* = 0.792), resting memory CD4 T cells (*p* = 0.084), activated memory CD4 T cells (*p* = 0.925), follicular helper T cells (*p* = 0.738), gammadelta T cells (*p* = 0.521), resting natural killer (NK) cells (*p* = 0.441), activated NK cells (*p* (0.714), resting dendritic cells (*p* = 0.049), activated dendritic cells (*p* = 0.0636), resting mast cells (*p* = 0.958), activated mast cells (*p* = 0.978), eosinophils (*p* = 0.924), and neutrophils (*p* = 0.4).

Figure 2D shows the correlation among TIICs in the pathogenesis of OSCC. The most interesting findings from Figure 2D are the negative correlations between macrophage M0 and M2 and between macrophage M0 and M1; however, a positive correlation between macrophage M1 and M2 was observed. Apart from such important finding regarding the three subsets of macrophages, the combination of TIICs with the most obvious correlation was also found and summarized herein: for example, resting NK cells were significantly positively correlated with resting memory CD4 T cells (Pearson correlation value = 0.62); follicular helper T cells were significantly positively correlated with monocytes (Pearson correlation value = 0.55); macrophage M1 was significantly negatively correlated with macrophage M0 (Pearson correlation value = −0.74); and resting memory CD4 T cells were significantly negatively correlated with macrophage M0 (Pearson correlation value = −0.65). Supplementary Figure 1 used the Kaplan–Meier curves to show the prognostic values of 22 TIICs for the overall survival of OSCC. Among the 22 TIICs, only one type of TIICs (neutrophils) was significantly related to overall survival (*p* = 0.031), while the other TIICs were not significantly related to the prognosis of OSCC.

### Identification of Risk ISGs With Prognostic Values

**FIGURE 3:**
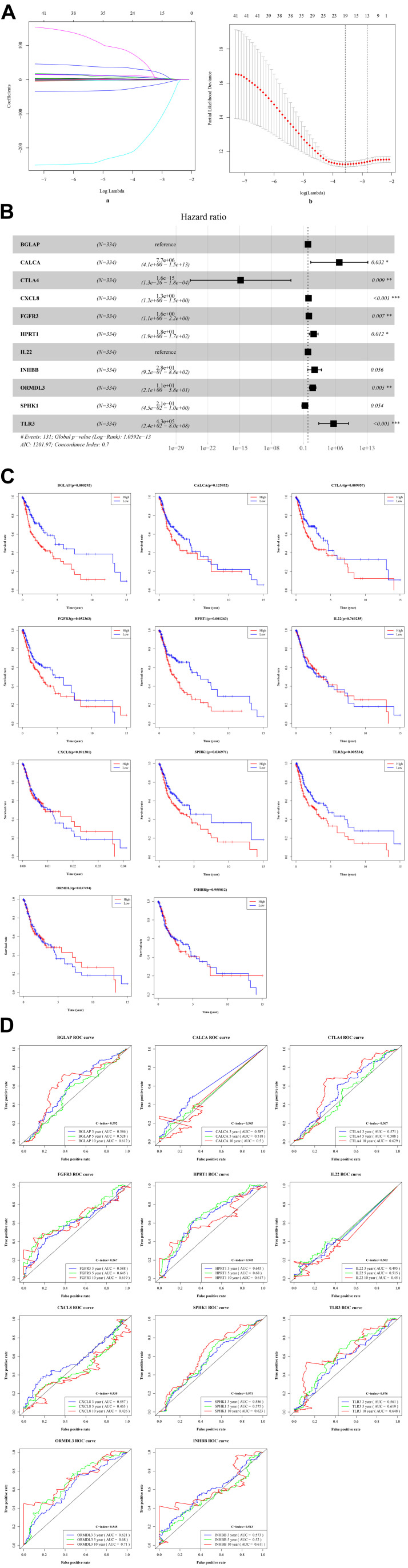
The identification of ISGs with significant prognostic values in OSCC. **(A)** Demographic and clinical feature selection using the LASSO binary logistic regression model. (a) LASSO coefficient profiles of the 42 gene features. A coefficient profile plot was produced against the log(lambda) sequence. All weights converge toward zero as the penalty parameter increases. Vertical line was drawn at the value selected using fivefold cross-validation, where optimal lambda resulted in five features with non-zero coefficients. (b) Optimal parameter (lambda) selection in the LASSO model used fivefold cross-validation via minimum criteria. The partial likelihood deviance (binomial deviance) curve was plotted versus log(lambda). Dotted vertical lines were drawn at the optimal values by using the minimum criteria and the 1 standard error (SE) of the minimum criteria (the 1-SE criteria). **(B)** The forest plot with hazard ratio (HR) for the 11 risk ISGs. HRs above one indicates that a gene is positively associated with the event probability and thus negatively with survival time. The box size is based on precision, and the x-axis has a logarithmic scale. A bigger box size represents a more precise confidence interval (95% CI). **(C)** The Kaplan–Meier curves of the 11 risk ISGs. Red lines represent high expression group, while blue lines represent low expression group. The upper line indicates the higher survival rate, while the lower line indicates the lower survival rate. **(D)** The time-dependent receiver operating curve (ROC) is generated for the survival prediction of 11 risk ISGs. The time-dependent (3-, 5-, and 10-year) area under curve (AUC) and C-index for each risk ISG are respectively labeled in the lower right corner of each gene’s ROC curve.

By performing the univariate analysis, 42 ISGs with *p* < 0.01 were identified (Supplementary Table 1). These 42 gene features were reduced to 19 genes (Figure 3A) by performing the LASSO regression analysis. As shown in Figure 3A (b), when the log (Lambda) = 19, the partial likelihood deviance reached the lowest value. Regarding the 19 genes, multivariate survival analysis was performed, and thus, 11 risk ISGs were identified: CXCL8, TLR3, IL22, ORMDL3, FGFR3, CTLA4, HPRT1, BGLAP, CALCA, SPHK1, and INHBB (Supplementary Table 2). The forest plot of these 11 risk ISGs is shown in Figure 3B. In addition, Kaplan–Meier curves shown in Figure 3C shows that six ISGs (e.g., BGLAP, CTLA4, HPRT1, ORMDL3, SPHK1, and TLR3) were found to be significantly associated with the survival rate of OSCC, showing that the low expression of all these six ISGs has higher survival rate compared with the high expression group. Figure 3D uses the time-dependent receiver operating characteristic (ROC) curves to assess the prediction accuracy of the 11 risk ISGs signature. Supplementary Table 3 shows the C-index values and time-dependent area under the curve (AUC) values of 11 risk ISGs in the ROC curves. In general, the ROC curve showed good performance in survival prediction as for the almost all 11 ISGs’ 10-year overall survival (AUC > 0.5 and C-index > 0.5). Specifically, taking ORMDL3 as an example, the AUC values of ORMDL3 gene were shown to be 0.621, 0.68, and 0.71, respectively, for 3, 5, and 10 years. Taking CTLA4 as another example, the AUC values of CTLA4 gene were shown to be 0.571, 0.508, and 0.629, respectively, for 3, 5, and 10 years.

**FIGURE 4:**
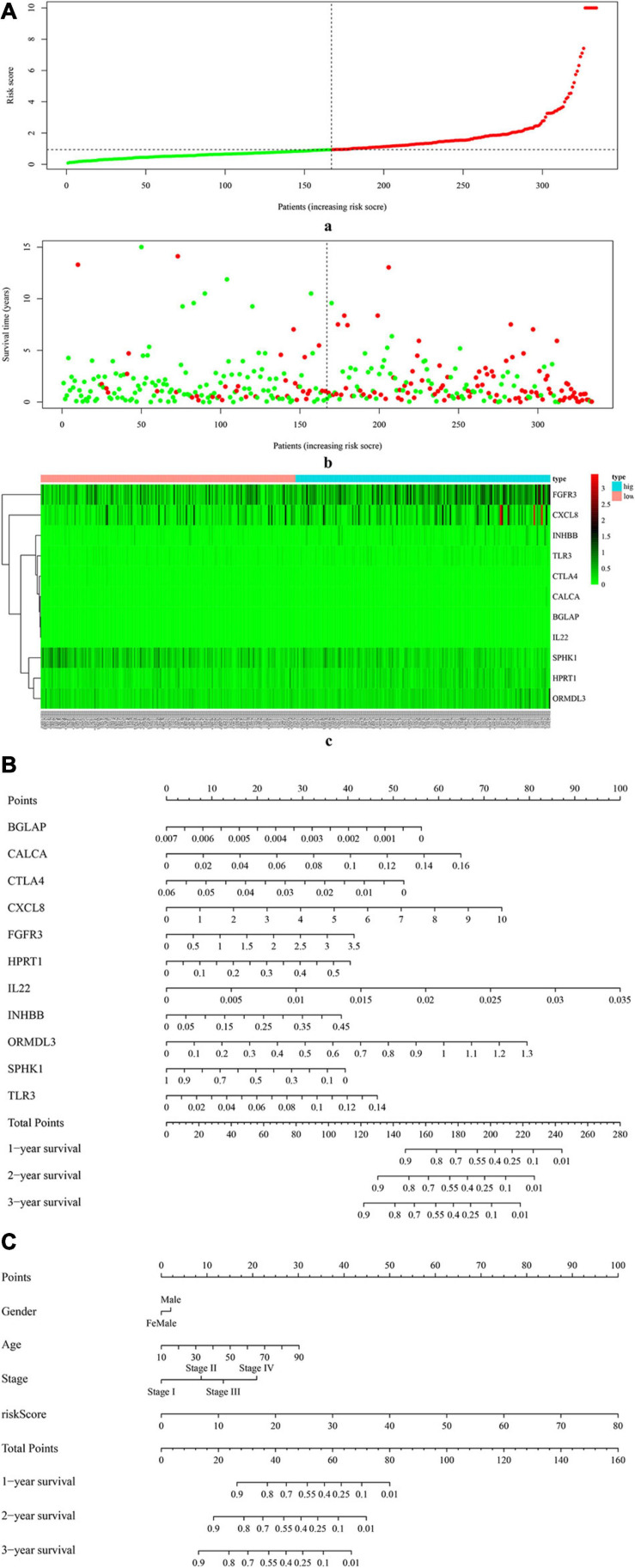
The correlation between 11 risk ISGs and survival of OSCC. **(A)** Prognostic risk score model analysis of 11 risk ISGs in OSCC patients. (a) The distribution of the risk scores in the OSCC sample. The x-axis represents the sample number of OSCC samples; the y-axis represents the risk score corresponding to the sample number. The green part of the curve that is lower than the median value represents the comparatively lower risk of survival, and the red part of the curve that is higher than the median value represents the higher risk of survival. (b) The distribution of patients’ survival status in the OSCC sample. The graph is drawn based on whether the final state of each OSCC patient during the follow-up period is alive or dead. The abscissa represents the risk score of OSCC patients, and the ordinate represents survival time. Each dot represents an OSCC patient: the red dot represents that the OSCC patient is dead at the final day of follow-up period, whereas the green dot represents that the OSCC patients is still alive at the final day of follow-up period. (c) The 11 risk ISGs expression profiles of patients in the low- and high-risk groups of OSCC samples. Each column represents a OSCC sample, and each row represents the expression profile of each gene within the 11 risk ISGs. The expression levels of the 11 risk ISGs in OSCC samples are shown in different colors, which transition from green to red. The abscissa represents the 148 OSCC samples: pink represents the OSCC samples with low risk of survival, and sky blue represents the OSCC samples with the high risk of survival. The ordinate represents the expression levels of TIICs in each OSCC sample. In the color bar, green represents the low expression of 11 ISGs in samples, red represents the high expression of 11 ISGs in samples, and black represents that the ISGs were not expressed in the OSCC samples, meaning the expression level was zero. **(B)** Nomogram for predicting 1-, 2-, and 3-year probabilities of overall survival in OSCC patients according to the expression level of 11 risk ISGs. The total score was 0–280. Total score of an individual patient is calculated and merged based on each variable. A high score indicates a high risk of survival. A line is drawn upward to determine the score received for each variable value. The sum of these scores is located on the total points axis; then, a line is drawn downward to the survival axes to determine the likelihood of 1-, 2-, or 3-year overall survival. **(C)** Nomogram for predicting 1-, 2-, and 3-year probabilities of overall survival in OSCC patients according to the risk score as well as clinical characteristics of samples (e.g., gender, age, and pathological stage). Total score was 0–160. Total score of an individual patient is calculated and merged based on each variable. A high score indicates a high risk of survival. A line is drawn upward to determine the score received for each variable value. The sum of these scores is located on the total points axis; then, a line is drawn downward to the survival axes to determine the likelihood of 1-, 2-, or 3-year overall survival.

Figure 4A (a) shows the risk score of all OSCC samples, and Figure 4A (b) shows the survival status of each OSCC sample during the follow-up time. Thereby, two genes (i.e., FGFR3 and CXCL8) were found to be highly expressed in the high-risk group. Furthermore, Figure 4B uses a nomogram to predict the probability of 1-, 2-, and 3-year overall survival according to the expression pattern of 11 risk ISGs. By adding up the points identified on the point scale for each variable, the total score on the bottom scale shows the probability of survival. Supplementary Tables 4–8 respectively show the clinical characteristics of the OSCC samples collected from the TCGA database, ICGC database, and three datasets (i.e., GSE41613, GSE42743, and GSE75538). Based on the information obtained from Supplementary Tables 4–8, the clinical features-related nomogram was plotted and shown in Figure 4C.

### Identification of Two Molecular Subtypes of OSCC by Using Deep Learning Framework

**FIGURE 5:**
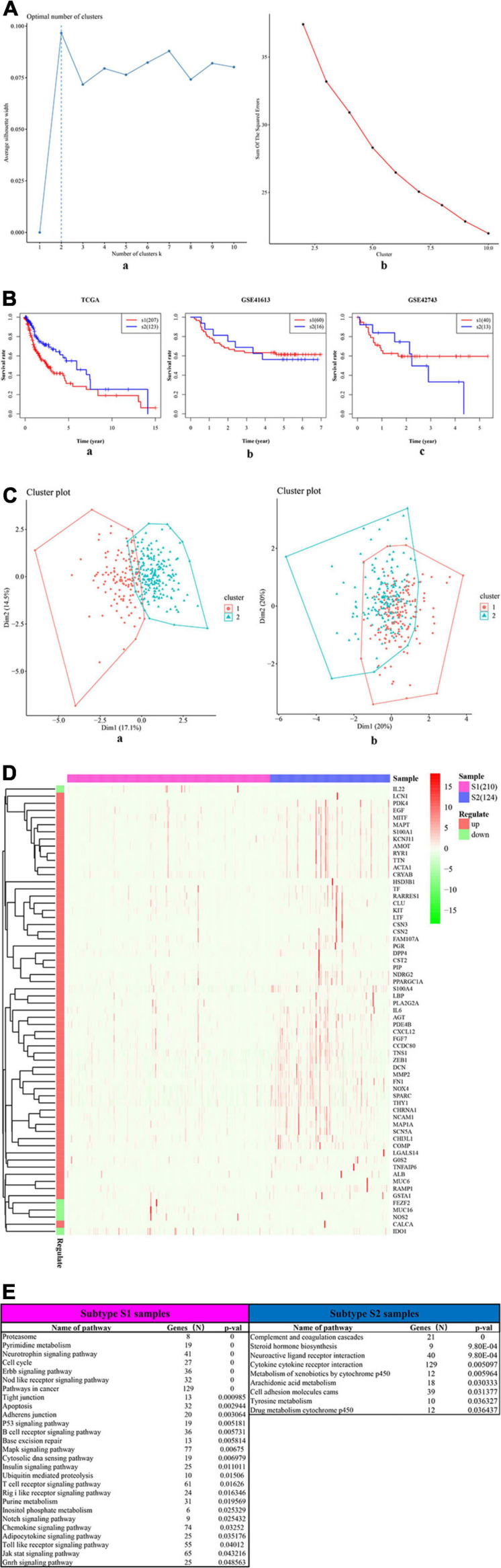
The two survival subtypes of OSCC differentiated by deep learning-based model. **(A)** The clustering evaluation plots drawn by using the silhouette index (a) and Calinski–Harabasz criterion (b), respectively. (a) The silhouette index values for each number of clusters. The abscissa represents the number of clusters, and the ordinate represents the value of average silhouette width. The plot shows that the highest value of average silhouette width occurs at two clusters, suggesting that the optimal number of clusters is two. (b) The Calinski–Harabasz criterion values for each number of clusters. The abscissa represents the number of clusters, and the ordinate represents the value of sum of squared errors. The plot shows that when the clusters = 2, the sum of squared errors arrived at the highest values, indicating that the optimal number of clusters is two. **(B)** The survival differences between two subtypes, respectively, for the TCGA (a) and the two external confirmation cohorts [GSE41613 (b) and GSE42743 (b)]. The red curve represents the subtype Sub1, and the blue curve represents the subtype Sub2. The abscissa represents the follow-up time that was calculated in the number of year, and the ordinate represents the survival rate. The upper line indicates the higher survival rate, while the lower line indicates the lower survival rate. **(C)** The two-dimensional (2D) scatter plots showing the comparisons between deep-learning based method (a) and PCA-based method (b). The abscissa represents the dimension 1 (Dim1), while the ordinate represents the dimension 2 (Dim2). (a) The scatterplot by using top 100 principal components as supposed to 100 hidden nodes in deep learning, followed by the subsequent Cox-PH and K-means clustering. (b) The scatterplot by using deep learning based method. **(D)** The heatmap shows the expression patterns of ISGs–DEGs in two types of samples, i.e., samples of subtype Sub1 and Sub2. The abscissa represents OSCC samples: the rose-red samples represent 210 subtype Sub1 samples, and blue samples represent 124 subtype Sub2 samples. The ordinate represents the ISGs that were also DEGs dysregulated between the two subtypes. Pink represents the upregulated DEGs, and emerald green represents the downregulated DEGs. **(E)** The top list of signaling pathways shows the significantly enriched cancer-related signaling pathways that were enriched by 1,180 ISGs in the samples of subtype Sub1 and Sub2, respectively.

The univariate Cox-PH regression model revealed 10 features, which were subjective to K-means clustering with cluster number K ranging from 2 to 10. Figure 5A (a) and (b) show the clustering evaluation plots by using the silhouette index and Calinski–Harabasz criterion, respectively. Figure 5A indicates that *K* = 2 was the optimal number of clusters with the best evaluation scores for both metrics. Afterward, the two subtypes identified in the above analysis were used as labels to construct an SVM classification model. The parameter settings used in the SVM algorithm and the subsequent autoencoder algorithm are shown in Supplementary Tables 9, 10, respectively. The classification effects of the SVM model were evaluated by assessing C-index to assess the accuracy of the survival subtype predictions and Brier score to calculate the error of the model fitting on survival data. The evaluation effects of the model in TCGA (e.g., training data, test data, and all data), and four independent confirmation sets (i.e., GSE41613 dataset, GSE42743 dataset, GSE75538 dataset, and ICGC data) are shown in Supplementary Table 8.

Figure 5B shows the significant survival differences for the TCGA (a) and the two external confirmation cohorts [GSE41613 (b) and GSE42743 (c)]. For the TCGA set, subtype Sub1 received a lower survival rate compared with the subtype Sub2, showing that subtype Sub1 is more aggressive and represents the higher survival risk. Regarding the two confirmation sets (GSE41613 and GSE42743), the same trend was observed within the beginning years of the observed time (GSE4163, 0–3 years; GSE42743, 0–2 years), and the opposite trend was observed for the later years of observed time. In addition, the performance of the model was compared by using an alternative approach—PCA. Figure 5C shows the scatter plots of DL-based model (a) and PCA-based model (b). Obviously shown in Figure 5C, the two subtypes of OSCC can be clearly divided by using the DL model [Figure 5C (a)]; by contrast, the two subtypes of OSCC cannot be clearly divided by using the PCA model [Figure 5C (b)]. By checking the C-index and *p*-value data shown in Supplementary Table 11, it can be found that the PCA model produced a lower C-index (0.69) as compared with the DL model (0.77); although the PCA approach can also yield a significant log-rank *p*-value (5.36E-18) in detecting the survival subgroups, the *p*-value produced by the PCA model is still much less significant than that by the DL model (4.91E-22).

### Identification of the Difference Between Varying Subtypes

**FIGURE 6:**
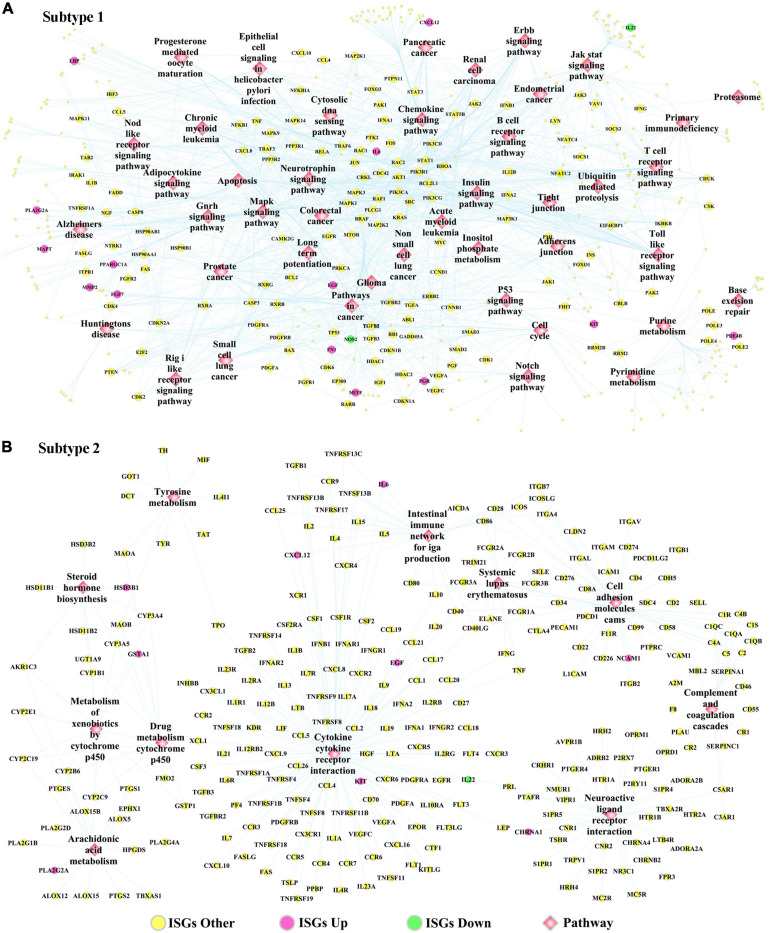
The ISGs-pathways interaction network in OSCC samples of panel **(A)** subtype Sub1 and panel **(B)** subtype Sub2. The rose-red round nodes represent ISGs that were DEGs upregulated between two subtypes of OSCC, the emerald round nodes represent ISGs that were also DEGs downregulated between two subtypes of OSCC, the yellow round nodes represent ISGs that were not DEGs dysregulated between two subtypes of OSCC, and the diamond nodes represent the signaling pathways targeted by ISGs.

The DEGs dysregulated between two subtypes are shown in Supplementary Table 12, ranked by the ascending order of *p*-value. Figure 5D used a heatmap to show the expression pattern of DEGs in the samples of two subtypes. By performing the functional enrichment of 1,180 ISGs in two subtypes, the significantly enriched pathways were selected. Among the enriched pathways, the pathway that was obviously not related to oral cancer were deleted, and the pathways potentially related to OSCC were retained and shown as Figure 5E. As seen from Figure 5E, the ISGs in subtype Sub1 were mainly enriched in tumor-infiltrating immune cells-related pathways [e.g., B cell receptor (BCR) signaling and T cell receptor (TCR) signaling] and tumor progression-related pathways (e.g., cell cycle, apoptosis, p53, MAPK, Notch, chemokine, Toll-like receptor, and JAK-STAT), whereas the ISGs enriched in subtype 2 were mainly enriched in the metabolism-related pathways (e.g., metabolism of xenobiotics by cytochrome p450, arachidonic acid metabolism, and tyrosine metabolism). Based on the significant pathways listed in Figure 5E, the ISGs-pathways interaction network for subtype Sub1 and S2 are shown in Figures 6A,B, respectively.

**FIGURE 7:**
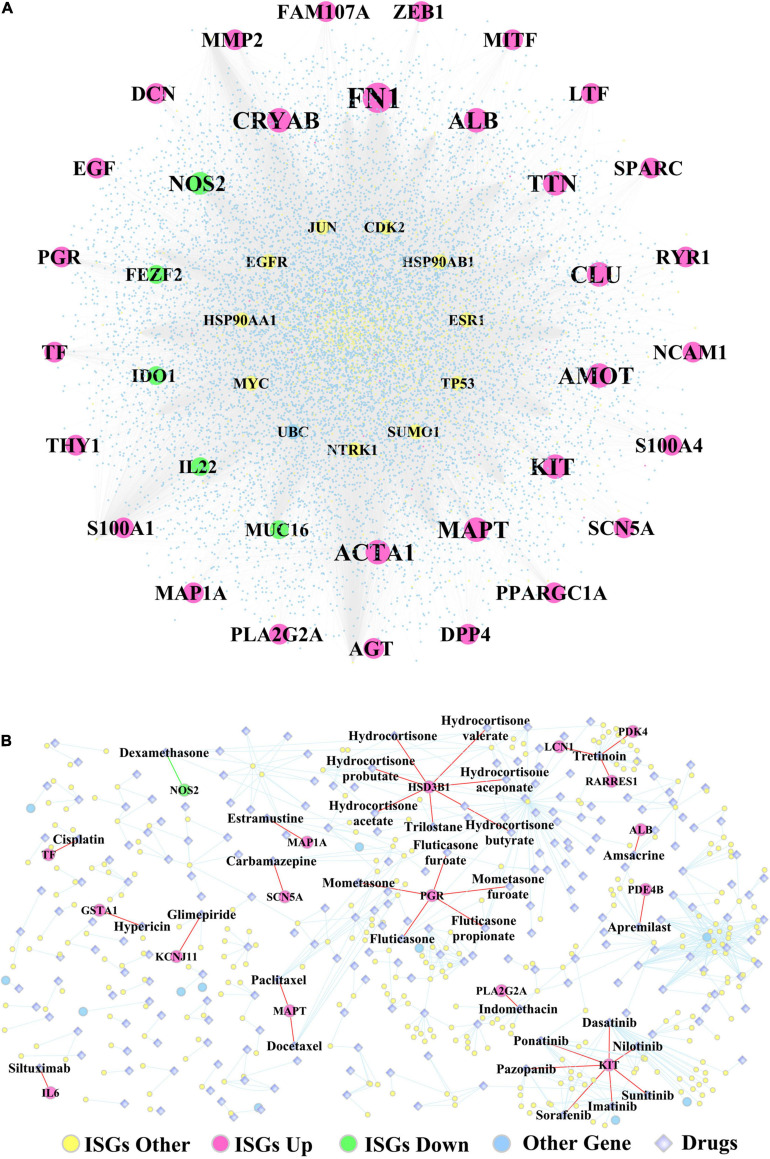
**(A)** The PPI network constructed by ISGs and **(B)** the ISGs-target drugs regulatory network. The rose-red round nodes represent the ISGs that were DEGs upregulated between two subtypes of OSCC, the emerald round nodes represent the ISGs that were DEGs downregulated between two subtypes of OSCC, the yellow round nodes represent the ISGs that were not DEGs, the sky blue round nodes represent the genes that were not ISGs, and the diamond nodes represent the drugs targeted by ISGs.

Supplementary Table 13 lists the fold change, log_2_(fold change), *p*-values, and adjusted *p*-values of DEGs, which were differentiated between the two subtypes of OSCC samples. In addition, Figure 7A shows the ISGs–DEGs-related PPI network, and the topological characteristics of the top 20 DEGs in this network are shown in Supplementary Table 14. The PPI network identified several hub genes that play critical roles by targeting the greatest number of other genes, for example, the ISGs-upregulated DEGs (e.g., FN1, ALB, ACTA1, TTN, MAPT, MMP2), the ISGs-downregulated DEGs (e.g., NOS2, MUC16, IDO1, IL22, and FEZF2), and ISGs–non-DEGs (e.g., NTRK1, JUN, TP53, MYC, EGFR, HSP90AA1, and ESR1). Furthermore, the ISGs–target drugs interaction network shown in Figure 7B shows that NOS2—the only ISG-downregulated DEGs mapped in this network—was targeted by the drug dexamethasone. In addition, the drug Tretinoin was found to target three ISGs (i.e., LCN1, PDK4, and RARRES1), which were upregulated in subtype Sub1; the ISG-HSD3B1, which was upregulated in subtype Sub1, was found to be targeted by several drugs including hydrocortisone, hydrocortisone valerate, hydrocortisone aceponate, hydrocortisone butyrate, hydrocortisone probutate, hydrocortisone acetate, and trilostane; and the ISG-PGR, which was upregulated in subtype Sub1 was found to be targeted by several drugs including fluticasone, fluticasone furoate, mometasone, mometasone furoate, and fluticasone propionate.

## Discussion

The main findings of the present study include several key aspects, such as the correlation between tumor-infiltrating immune cells, identification of 11 ISGs that were significantly related to the overall survival of immunocompromised OSCC patients, signaling pathways differentiating the two key molecular subtypes of OSCC, and the identification of drugs that were targeted by ISGs.

The interaction between immune cells has been explored by many previous studies; however, the synergistic or antagonistic impact of specific immune cells on tumor immunology varies among different cancer types and therefore remains ill defined. Existing evidence strongly supports the findings of the present work; however, contradictory findings have also been reported. Heterogeneity in tumor-intrinsic type, tumor microenvironment (TME), and the tissue specificity in various cancer types may be plausible causes. For instance, considering macrophage M2 phenotype that displays immunosuppressive functions and interplays with other immune cells (e.g., NK cells, Treg, CD8 T cells, and neutrophils) showed a negative correlation with activated NK cells (*p* = −0.04) in our study. A study by Nuñez noted that M2 macrophages can restrain NK cell activation and effector functions, thereby resulting in suppression of IFN-γ production by NK cells with impaired cytotoxic capacity and degranulation ability (Nuñez et al., 2018). Here, a negative correlation between macrophage M2 and Treg in OSCC (*p* = −0.22) was noted, which is contradictory to previous findings shown in other cancer types. A recent study regarding renal cell carcinoma reported synergistic effects between macrophage M2 and Tregs, showing that macrophage M2 played its protumor and immunosuppressive role by releasing cytokines, thereby activating and recruiting Tregs (Davidsson et al., 2020). Another study investigating laryngeal cancer also showed that the combination of a high number of M2 macrophage and Tregs indicated worse prognosis (Sun et al., 2017). Furthermore, the present study showed a positive correlation between macrophage M2 and CD8 T cells (*p* = 0.22), which is contradictory with previous evidence showing that macrophage M2-like TAMs can suppress the recruitment and function of CD8 + T cell, thereby favoring tumor immune escape and driving tumor progression (Dannenmann et al., 2013; Peranzoni et al., 2018; Quaranta and Schmid, 2019). Moreover, the present study showed a positive correlation between macrophage M2 and neutrophils (*p* = 0.16), similar to earlier reported findings showing both M2-like TAMs and TANs can exert immunosuppressive and protumoral functions and also share overlapping pathways to crosstalk with T cells (Kim and Bae, 2016). Both types of cells can work in a partnership to modulate tumor immunity, thus have been regarded as “partners in crime” (Kumar et al., 2018). Taken together, the research regarding the crosstalk between immune cells in oral cancer immunology is still in progress and needs further investigation.

Another important finding regarding TIICs analysis is the correlation between three phenotypes of macrophages. The current research showed a positive correlation between M1 and M2 and negative correlations between macrophage M0 and M2 and between M0 and M1. Activated macrophages are classified as two subsets with the entirely different functions: M1 macrophages, which are proinflammatory and antitumoral, and M2 macrophages, which are anti-inflammatory and protumoral (Jayasingam et al., 2020). The current research found a positive correlation between M1 and M2, which is contradictory to some previous literature, which is quite variable. Some studies found negative correlation between M1 and M2 by showing that the promotion of M2 macrophage polarization could suppress the M1 macrophage polarization, and vice versa, the inhibition of M2 macrophage polarization could promote the M1 macrophage polarization (Abdelaziz et al., 2020; Peng et al., 2020). However, a previous study regarding breast cancer found a weak correlation between M1 and M2 macrophage densities in central tumor tissue of breast cancer (Schnellhardt et al., 2020). One reason for such contradictory results might be because of the different cancer types studied. Notably, research investigating the correlation between M1 and M2 is limited; however, much of the current cancer research has focused on investigating the M1/M2 ratio and its relationship with prognosis. It has been well concluded that the high infiltration of M1 macrophages and the low infiltration of tumor-infiltrating M2 macrophages are associated with better prognosis (Jayasingam et al., 2020). Another study based on TCGA database of oral cancer data also obtained the same conclusion by showing that a high M2/M1 ratio indicated poor overall survival in human primary oral cancers (Dan et al., 2020). In addition, the current research also found a negative correlation between the non-activated M0 macrophage and activated macrophages (M1 and M2); however, up until now, there is no research evidence showing the correlation between naive non-activated M0 and polarized macrophages M1/M2 phenotype. We speculate that high infiltration of M0 might inhibit the polarization of M0 to M1/2, and vice versa, the high infiltration of M1/M2 might promote the polarization of M0 to M1/2, which warrants validation by future work. Another observation showed a high expression of naive M0 in cancer samples compared with healthy control samples and significantly low expression of M1 macrophage in cancer samples compared to healthy control samples, which in turn confirmed a negative correlation between M0 and M1.

The role of the identified key genes and signaling pathways in regulating immunosuppression of OSCC has been largely validated in previous experimental studies. The survival analysis showed the 11 ISGs (i.e., CXCL8, TLR3, IL22, ORMDL3, FGFR3, CTLA4, HPRT1, BGLAP, CALCA, SPHK1, and INHBB) to be significantly correlated with overall survival in OSCC. Existing evidence has shown that almost all these genes are linked to inhibition of the immune response during the oncopathogenesis in OSCC and are also related to survival in oral cancer. The overexpression of C-X-C motif chemokine ligand 8 (CXCL8, also called IL-8) in OSCC has been related to poor prognostic outcome due to its promoting effect on the generation and infiltration of CD163-positive M2 type tumor-associated macrophages, which can support and exacerbate the immunosuppression by tumor-infiltrating T cells (Hosono et al., 2017). Toll-like receptor 3 (TLR3) stimulation of oral cancer cells can cause tumor progression (Urban-Wojciuk et al., 2019), which is evidenced by the fact that the stimulation of TLR3-expressing oral cancer cells lines (buccal OC2 cancer cells) was found to lead to tumor progression via the production of immunosuppressive factors (Chuang et al., 2012). IL-22 is a cytokine with tumor-promoting properties, which can mediate the attraction of immunosuppressive immune cells and regulate the release of pro- and anti-inflammatory cytokines (Voigt et al., 2017). ORMDL sphingolipid biosynthesis regulator 3 (ORMDL3) can encode a protein that belongs to a family of transmembrane proteins of the endoplasmic reticulum and is found to be involved in the activation of the immune system by regulating calcium signaling (Carreras Sureda, 2014). Fibroblast growth factor receptor 3 (FGFR3), a member of the fibroblast growth factor receptor (FGFR) family (FGFR 1–4), has been found to be frequently overexpressed in OSCC (Koole et al., 2016). Using an FGFR inhibitor to block the binding between FGFR and its ligand FGF was found to remodel the immune microenvironment of tumors by inducing new T-cell responses and in turn work synergistically with PD1 inhibitor in promoting antitumor immunity (Palakurthi et al., 2019). However, a previous study obtained conflicting results showing that the overexpression of FGFR3 protein was not related to overall survival or disease-free survival in OSCC (Koole et al., 2016).

In addition, ISGs with prognostic values also included CTLA4, HPRT1, BGLAP, CALCA, SPHK1, and INHBB. The blockade of cytotoxic T lymphocyte-associated antigen 4 (CTLA4) was demonstrated to decrease the number of immunosuppressed cells [e.g., myeloid-derived suppressor cells (MDSCs) and M2 macrophages] and further enhance the activation of T cells, thereby suggesting a novel therapeutic target for treating OSCC (Yu et al., 2016). The overexpression of hypoxanthine phosphoribosyltransferase 1 (HPRT1) can contribute to the formation of an immunosuppressive tumor microenvironment by significantly reducing the activation of immune cells (B cells, CD8 + T cells, CD4 + T cells, macrophages, and neutrophils) (Townsend et al., 2019). There is still no research reporting the involvement of bone gamma-carboxyglutamate protein (BGLAP, also named osteocalcin) in oral cancer. The uncarboxylated form of osteocalcin (GluOC) was found to suppress tumor growth of melanoma through immunostimulatory effects via increasing T-cell proliferation and promoting the interferon-γ production (Hayashi et al., 2017). Calcitonin-related polypeptide alpha (CALCA, also named as CGRP) was shown to suppress the immune reactions by inhibiting the production of tumor necrosis factor-α and interferon-γ by T helper type 1 cells via elevating intracellular cAMP levels (Kawamura et al., 1998) and impairing the capacity of Langerhans cells in stimulating the T cells proliferation (Hosoi et al., 1993). Sphingosine kinase 1 (SPHK1)-involved SphK–S1P–S1PR signaling axis was found to mediate immunosuppressive effects by affecting lymphocyte trafficking, activating innate immune cells and inflammation, and directing T-cell differentiation (Chi, 2011). The inhibin subunit beta B (INHBB) gene encodes a member of the transforming growth factor-beta (TGF-β) superfamily. TGF-β has been well accepted as an immunosuppressive cytokine in cancer progression, which can suppress the expression of chemokine receptor CXC motif 3 (CXCR3) in CD8 + T cells and thus limit the tumor trafficking (Gunderson et al., 2020).

Apart from the prognosis-related ISGs described above, the tumor-infiltrating immune cells-related pathways and tumor progression-related pathways (TCR, BCR, p53, JAK-STAT, MAPK, and Notch) enriched in subtype Sub1 cancer samples were identified to predict worse survival in OSCC. Past work has highlighted the potential functions of these worse prognosis-related pathways in the immunosuppression in OSCC. The prognostic values of tumor-infiltrating T cells and B cells in OSCC have been widely accepted by cancer immunology researchers (O’Higgins et al., 2018; Taghavi et al., 2018). TCRs complex (e.g., costimulatory and coinhibitory receptors)-initiated TCR signaling has been demonstrated to play significant roles in regulating immune response, particularly in terms of the activation, differentiation, proliferation, and survival of T cells; thus, it might be a therapeutic target for immune suppression (Hwang et al., 2020). B cell receptor (BCR)-mediated calcium flux was found to play a crucial role in immunosuppression by promoting the secretion of an immunosuppressive cytokine—IL-10 in B cells (Klinker and Lundy, 2012). The mutant p53 was found to promote the development of tumorigenesis by interfering with the function of the cytoplasmic DNA sensing machinery pathway cGAS-STING-TBK1-IRF3 and further suppressing the innate immune response (Cooks et al., 2018). The transduction of Janus kinase-signal transducer and activator of transcription (JAK-STAT) signaling can lead to the production of the protumor cytokines [e.g., IL-1, IL-17, IL-10, TGF-β, vascular endothelial growth factor (VEGF)], which can promote tumor immunogenicity and inhibit the antitumor immune response (Owen et al., 2019). Mitogen-activated protein kinase (MAPK) signaling has been shown to inhibit the expression of negative immune checkpoints [e.g., programmed death-ligand 1 (PD-L1) and cytotoxic T-lymphocyte-associated protein 4 (CTLA-4)] and T-cell costimulatory molecules [e.g., tumor necrosis factor receptor superfamily member 4, and 9 (TNFRSF4, TNFRSF9)]; therefore, the inhibition of MAPK signaling is promising for combined use with T-cell-dependent immunotherapy for antitumor treatment (Kumar et al., 2020). The Notch pathway was found to be a multifaceted regulator of immune-suppressive cells—myeloid-derived suppressor cells (MDSCs); thus, inhibiting MDSCs by targeting the Notch pathway might be a novel immunotherapeutic strategy of cancer treatment (Hossain et al., 2018). In addition, the Notch pathway was shown to play a pivotal role in maintaining the stemness of cancer stem cells in tongue cancers and has prognostic value in OSCC (Upadhyay et al., 2016).

It is worthwhile to note that the signaling pathways enriched in subtype Sub1 and Sub2 were quite different, and metabolic pathways were mainly enriched in subtype Sub2. It has been well demonstrated that the dysregulated metabolic pathways of cancer cells could result in enhanced nutrient uptake, decreased oxygen (hypoxia), and increased acidity of extracellular milieu, and a shortage of nutrition, and the upregulation of protumor metabolite production (Jiang et al., 2020). All these alterations contribute greatly to an immunosuppressive TME, thereby impairing the antitumor immune response and further promoting the tumor progression (Biswas, 2015). Targeting metabolic pathways is therefore an immunotherapeutic approach to inhibit the tumor progression via restoring the TME environment (Shi et al., 2020). In the present study, several signaling pathways related to metabolism were mainly enriched in subtype Sub2, for example, tyrosine metabolism, cytochrome p450 metabolism, and arachidonic acid metabolism. The downregulation of tyrosine metabolism pathway-related genes (HPD, HGD, GSTZ1, and FAH) was observed in hepatocellular carcinoma and indicated poor prognosis (Nguyen et al., 2020); however, investigation of dysregulation of tyrosine metabolism pathways in OSCC is lacking. In addition, the inhibitor of tyrosine kinase, Imatinib, was shown to play both immunostimulatory and immunosuppressive role in the tumor immunology, an immunostimulatory role by stimulating the ability of dendritic cells and NK cells, and an immunosuppressive role by inhibiting the proliferation of T cells (Nishioka et al., 2011). In terms of the cytochrome p450 (CYP) metabolism pathway, the polymorphisms of CYP-involved genes (e.g., CYP26B1, CYP1A1, CYP2A6, and CYP2E1) have been found to activate areca nut (AN)-derived nitrosamines, thereby significantly increasing the susceptibility to tobacco-induced oral cancer (Lin et al., 2013). Considering the arachidonic acid metabolism (AAM) pathway, its mutation was shown to suppress the progression of oral cancer by downregulating it downstream PI3K-Akt pathway and was also predicted to indicate a better disease-free survival (Biswas et al., 2014). Together, these reports lend support to our findings.

Considering targeting drugs, the present study found that dysregulated ISGs that were differentially expressed between subtypes Sub1 and Sub2 were mapped to some target drugs, e.g., downregulated ISG-NOS2 targeting dexamethasone and target drug Tretinoin targeting three upregulated ISGs (e.g., PDK4, LCN1, and RARRES1). The overexpression of the inducible NO synthase including INOS and NOS2 has been documented to predict poor survival outcome in multiple cancers, based on the its involvement in immunosuppression by altering the tumor microenvironment and resulting in the resistance to the inhibitor of immune-checkpoint genes (Ekmekcioglu et al., 2017). However, the expression level and regulating function of NOS2 varies a lot depending on the cancer type; for example, the downregulation of NOS2 in the subtype 1 of OSCC was associated with the worse survival in the present study. The target drug dexamethasone has been found to mediate T cells-mediated immunosuppression by inhibiting the expression of inducible NOS2 and upregulating the expression of the immune-checkpoint gene CTLA-4 (Korhonen et al., 2002; Giles et al., 2018); therefore, using an antagonist of dexamethasone might be an immunotherapy approach for improving the survival outcome in immunocompromised patients with OSCC. For another example, the drug tretinoin (also named as all trans retinoic acid) targeting three ISGs (PDK4, ICN1, RARRES1) was shown to dramatically reduce the presenter of immature myeloid cells, which promoted immunosuppression by increasing the production of reactive oxygen species, and thus contributed greatly to tumor progression (Kusmartsev et al., 2003; Wu et al., 2019). As one of the genes targeting tretinoin, the overexpression of pyruvate dehydrogenase kinase 4 (PDK4) was found to promote tumor cells proliferation and invasion by negatively regulating the IL-10 expression in macrophages and thus indicated poor prognosis (Atas et al., 2020; Na et al., 2020), which was in accordance with results obtained in the present study. As another gene targeting tretinoin, the Lipocalin 1 (LCN1) was found highly expressed in cholangiocarcinoma and its overexpression indicated poor survival outcome (Tian et al., 2018); however, its involvement in tumor immunosuppression has not yet been explored. Another gene targeting tretinoin, the retinoic acid receptor responder 1 (RARRES 1) gene, was found to play tumor suppressive function by negatively regulating metastasis (Huebner et al., 2017); however, its immunological function in oral cancer has not yet been researched. The target drugs highlighted through deep learning merit further experimental research.

Additionally, the potential applications of the present research findings in precision medicine need to be highlighted. In the past decade, much cancer research has been largely focused on identifying certain critical genetic/epigenetic biomarkers involved in cancers (Tao et al., 2017; Brenner, 2019); however, stratifying cancer patients according to the genetic biomarkers-defined subgroups can be of high clinical value. Cancer patients’ response to the same therapeutic treatment is notably variable. Some show worse survival, while others show better outcomes. There is therefore a strong necessity to define the molecular subtypes of cancer patients and their association with prognosis (Téllez-Gabriel et al., 2013; Kim et al., 2019). Cancer precision treatment paradigm should be shifted from a biomarkers-based paradigm to a subtyping-based paradigm, and tandem research has also shifted from identification of certain critical genetic targets to detection of subtype-specific genetic targets (Dienstmann et al., 2017; Torres and Grippo, 2018; Zhao S. et al., 2020). Based on such shift in the precision medicine paradigm, increasing number of studies apply molecular subtyping to establish treatment paradigms incorporating a precision medicine approach in cancer treatment (Zhao et al., 2018; Jiang et al., 2019; Lin et al., 2019). The present research aimed to provide OSCC subtypes that can enable treatment regimes targeting specific molecules and also refine prognosis. Here, we devised a deep learning-based model and provided a simplified approach to successfully stratify compromised OSCC patients into two different treatment arms according to their molecular subtypes with different prognosis. This subclassification approach provided in the present research has potential clinical transfer value by enabling drug selection guidance. In the context of the current research, treatment approach for the two subtypes can be varied: subtype 1 could be treated with drugs targeting the tumor-infiltrating immune cells-related pathways (TCR and BCR) and tumor progression-related pathways (p53, JAK-STAT, MAPK, and Notch); subtype 2 could be treated with metabolism-related pathways, particularly tyrosine metabolism, cytochrome p450 metabolism, and arachidonic acid metabolism. The development of such treatment strategy has the potential to improve OSCC outcomes.

It is essential to state the strengths and limitations of the present research clearly. The greatest strength is that deep machine learning, an artificial intelligence approach, was used in combination with bioinformatics for subtype discovery of oral cancer. The discovery of oral cancer subtypes can benefit targeted therapy for different subtypes of cancer patients and guide the precision medicine in oral cancer. Another strength is that this research was focused on investigating the involvement of immune suppression in OSCC from different aspects, such as immune cells, ISGs related to overall survival, target drugs of ISGs involved in OSCC, and the ISGs-involved signaling pathways used for differentiating two subtypes of OSCC. The present study has two main limitations. First, the findings shown in this study were obtained by computational analysis and not yet verified by performing molecular biology experiments. Second, the synergistic relationship between the ISGs-targeting drugs and commonly used chemotherapeutic drugs used for treating OSCC was not investigated by designing a drug synergy prediction model. However, the investigation of this topic could be regarded as another separate study and comprises our subsequent research plan.

The current findings have several future implications and potential clinical transfer value. First, the genetic mechanisms of ISGs OSCC identified in this study provide a theoretical basis and research direction for future studies. Future research can select the most critical ISGs for experimental studies aimed at investigating the regulating role of these ISGs in influencing oral cancer cell lines and immune cells (e.g., macrophages, neutrophils, and T cells) in cancer cells—immune cells coculture systems—and also investigating the effects of ISGs-targeting drugs on promoting the functions of immune cells and their antitumor effects. Studying the synergistic/antagonistic relationship between ISGs-targeting drugs and common chemotherapeutic drugs will help identify novel treatment strategies for immune–chemotherapy combination drugs. Second, the two subtypes of OSCC discovered by using deep machine learning could be beneficial for designing precision treatment plans for OSCC patients. OSCC patients with molecular subtype 1 could be specially treated with drugs with the role of targeting tumor immunology and progression, whereas the patients with subtype 2 could be particularly treated with drugs targeting metabolism. Such findings could guide the oral and maxillofacial clinicians to select the right target chemotherapeutic drugs and further increase the survival time of OSCC patients.

## Conclusion

Eleven immunosuppression genes (CXCL8, TLR3, IL22, ORMDL3, FGFR3, CTLA4, HPRT1, BGLAP, CALCA, SPHK1, and INHBB) were identified as significantly related to the prognosis of OSCC in immunosuppressed patients. A deep learning-based model was able to differentiate OSCC patients into two survival subtypes: a subtype with a lower probability of survival and a subtype with a higher probability of survival. Several immunosuppression-involved signaling pathways (e.g., T cell and B cell receptor signaling, p53, Notch, JAK-STAT, and MAPK) enriched in the aggressive subtype of OSCC suggested therapeutic targets, which could be valuable for treating OSCC in immunosuppressed patients and improving the overall survival in specific groups of patients.

## Data Availability Statement

Publicly available datasets were analyzed in this study. This data can be found here: GSE41613 (https://www.ncbi.nlm.nih.gov/geo/query/acc.cgi?acc=GSE41613), GSE42743 (https://www.ncbi.nlm.nih.gov/geo/query/acc.cgi?acc=GSE42743), and GSE75538 (https://www.ncbi.nlm.nih.gov/geo/query/acc.cgi?acc=GSE75538).

## Author Contributions

SL: conceptualization, funding acquisition, methodology, formal analysis, and writing—original draft. ZM and WG: methodology, formal analysis, and writing—original draft. AO, AA, GP, and WN: methodology, formal analysis, and writing—review and editing. XL and YD: data curation, formal analysis, methodology, resources, software, and visualization. HL: formal analysis and methodology. BL: formal analysis and methodology. VS: formal analysis and methodology. RZ: methodology and writing—review and editing. DZ, GS, HW, HX, and JZ: project administration, supervision, and writing—review and editing. All authors contributed to the article and approved the submitted version.

## Conflict of Interest

The authors declare that the research was conducted in the absence of any commercial or financial relationships that could be construed as a potential conflict of interest.

## Publisher’s Note

All claims expressed in this article are solely those of the authors and do not necessarily represent those of their affiliated organizations, or those of the publisher, the editors and the reviewers. Any product that may be evaluated in this article, or claim that may be made by its manufacturer, is not guaranteed or endorsed by the publisher.

## Footnotes

1
https://tcga-data.nci.nih.gov/tcga/

2
http://www.compgenome.org/TCGA-Assembler/

3
https://dcc.icgc.org/projects/ORCA-IN

4
https://www.ncbi.nlm.nih.gov/geo/query/acc.cgi?acc=GSE41613

5
https://www.ncbi.nlm.nih.gov/geo/query/acc.cgi?acc=GSE42743

6
https://www.ncbi.nlm.nih.gov/geo/query/acc.cgi?acc=GSE75538

7
http://www.disgenet.org

8
http://biokb.nb.org/HisgAtlas/

9
https://www.drugbank.ca/

10
https://cibersort.stanford.edu/

## Supplementary Material

Supplementary Figure 1 The Kaplan-Meier curves of the 22 TIICs. Red lines represent high expression group, while blue lines represent low expression group. The upper line indicates the higher survival rate, while the lower line indicates the lower survival rate. Among these 22 TIICs, only neutrophils have significant prognostic values in OSCC (*p* = 0.031), while the other 21 TIICs doesn’t have significant prognostic values (*p* > 0.05).

Supplementary Table 1 The 42 prognosis-related ISGs with *p*-value less than 0.01, ranked in the ascending order of *p*-value.

Supplementary Table 2 The 11 risk ISGs with the prognostic values in OSCC, ranked in the ascending order of *p*-value.

Supplementary Table 3 The C-index values and time-dependent AUC values of 11 prognosis-related ISGs shown in the ROC curves.

Supplementary Table 4 The clinical characteristics of OSCC samples (*n* = 317) from TCGA database.

Supplementary Table 5 The clinical characteristics of OSCC samples (*n* = 40) from ICGC database.

Supplementary Table 6 The clinical characteristics of OSCC samples (*n* = 97) from GSE41613 dataset.

Supplementary Table 7 The clinical characteristics of OSCC samples (*n* = 71) from GSE42743 dataset.

Supplementary Table 8 The clinical characteristics of OSCC samples (*n* = 14) from GSE75538 dataset.

Supplementary Table 9 The parameter settings of the SVM algorithm.

Supplementary Table 10 The parameter settings of the autoencoder algorithm.

Supplementary Table 11 The performance of the SVM classifier on training and test set in TCGA cohort, as well as the four external confirmation cohorts.

Supplementary Table 12 The model performance comparison between Deep learning-based model with the PCA-based model.

Supplementary Table 13 The all DEGs dysregulated between the two subtypes of OSCC samples, ranked by the ascending order of *p*-value.

Supplementary Table 14 The topological characteristics of the nodes in the ISGs-DEGs related PPI network. The listed nodes in this table include the top 20 ISGs-upregulated DEGs, top 20 ISGs-downregulated DEGs, and top 20 ISGs-non-DEGs.
