# Supplementary material for: Molecular Subtypes of Oral Squamous Cell Carcinoma Based on Immunosuppression Genes Using a Deep Learning Approach
Source: Front Cell Dev Biol. 2021 Aug 5;9:687245. doi: 10.3389/fcell.2021.687245 (PMC8375681; doi:10.3389/fcell.2021.687245)
Supplement: Supplementary Figure 1 — The Kaplan-Meier curves of the 22 TIICs. Red lines represent high expression group, while blue lines represent low expression group. The upper line indicates the higher survival rate, while the lower line indicates the lower survival rate. Among these 22 TIICs, only neutrophils have significant prognostic values in OSCC (p = 0.031), while the other 21 TIICs doesn’t have significant prognostic values (p > 0.05). [file Data_Sheet_1.docx]

Supplementary Material

# 1 Supplementary Figures and Tables

## Supplementary Figures


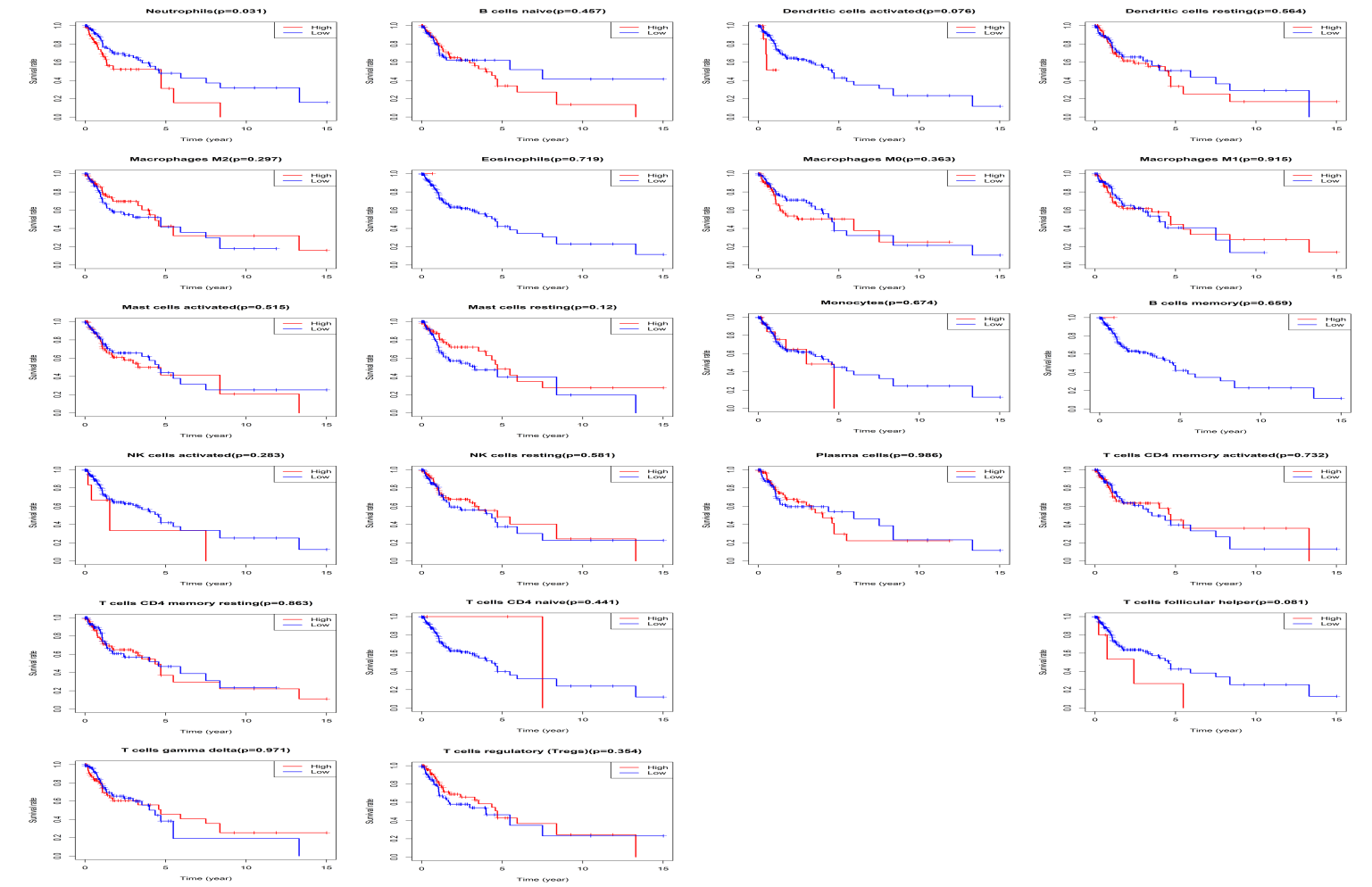


**Supplementary Figure 1.** The Kaplan-Meier curves of the 22 TIICs. Red lines represent high expression group, while blue lines represent low expression group. The upper line indicates the higher survival rate, while the lower line indicates the lower survival rate. Among these 22 TIICs, only neutrophils have significant prognostic values in OSCC (p=0.031), while the other 21 TIICs doesn’t have significant prognostic values (p>0.05).

## Supplementary Tables

**Table S1.** The 42 prognosis-related ISGs with p value less than 0.01, ranked in the ascending order of p value.

| **ISGs** | **HR** | **HR.95L** | **HR.95H** | **pvalue** |
| --- | --- | --- | --- | --- |
| **FGFR3** | 2.022995066 | 1.457952666 | 2.807024628 | 2.49E-05 |
| **NR4A1** | 2.030942634 | 1.440077672 | 2.864239939 | 5.37E-05 |
| **MUC16** | 2.346554547 | 1.546460235 | 3.560594782 | 6.09E-05 |
| **ORMDL3** | 18.24557816 | 3.982562698 | 83.58967519 | 0.00018435 |
| **CD200R1** | 1.05E+11 | 100549.5366 | 1.09E+17 | 0.000331856 |
| **HHLA2** | 1.19E+42 | 2.25E+17 | 6.33E+66 | 0.000851608 |
| **ALOX15** | 22.42302086 | 3.454417187 | 145.5504177 | 0.001118236 |
| **MAPK9** | 309.7355162 | 9.525701366 | 10071.28885 | 0.001243098 |
| **FOXA1** | 13.3210712 | 2.700893724 | 65.70082201 | 0.00147117 |
| **CXCL8** | 1.237047263 | 1.083558522 | 1.412278063 | 0.001648123 |
| **IL22** | 2.13E+48 | 1.33E+18 | 3.41E+78 | 0.001713309 |
| **CALCA** | 4808307705 | 3728.338369 | 6.20E+15 | 0.001899292 |
| **IRF2** | 47.48831343 | 3.789227887 | 595.1449688 | 0.002765567 |
| **CAMLG** | 532.4712009 | 8.493466851 | 33381.60786 | 0.002947262 |
| **NOS2** | 270.1900958 | 6.371811979 | 11457.13152 | 0.003405179 |
| **VDAC1** | 2.211839419 | 1.297603605 | 3.770206555 | 0.003529575 |
| **ATF2** | 16.18968219 | 2.463705797 | 106.3868136 | 0.003747965 |
| **TFCP2** | 59.30559037 | 3.726109078 | 943.9211186 | 0.003833233 |
| **MR1** | 26.40352009 | 2.77135987 | 251.5537158 | 0.004423522 |
| **SPHK1** | 0.140132567 | 0.035907278 | 0.546884578 | 0.004674187 |
| **GALNT7** | 18.89134822 | 2.427444138 | 147.0200825 | 0.004999248 |
| **AIMP1** | 66.277507 | 3.466362331 | 1267.238539 | 0.005341752 |
| **INHBB** | 84.52326443 | 3.696659559 | 1932.60486 | 0.005456612 |
| **TOB1** | 6.112236953 | 1.700844785 | 21.96522628 | 0.005541196 |
| **MAP2K1** | 11.88911289 | 2.06549266 | 68.43452319 | 0.005567128 |
| **ITCH** | 3.996037488 | 1.477635588 | 10.80666691 | 0.00634935 |
| **SEMA3A** | 34.05031624 | 2.686168239 | 431.6274832 | 0.00647869 |
| **MUC1** | 2.245253571 | 1.24783858 | 4.039916444 | 0.006960245 |
| **IRX3** | 23.17238552 | 2.326846552 | 230.7670226 | 0.007359805 |
| **PDE4D** | 74.34202198 | 3.130309516 | 1765.555835 | 0.007674694 |
| **PIGP** | 78875.9551 | 18.92074371 | 328814574.5 | 0.008017524 |
| **GOT1** | 8.880049814 | 1.765279311 | 44.67014608 | 0.008062267 |
| **STK16** | 652.4377443 | 5.38839447 | 78998.48695 | 0.008092352 |
| **TLR3** | 4637.999012 | 8.632148606 | 2491967.622 | 0.008488764 |
| **BECN1** | 30.08106735 | 2.378528863 | 380.4328916 | 0.008557161 |
| **BGLAP** | 1.35E-132 | 5.65E-231 | 3.24E-34 | 0.008610111 |
| **CTLA4** | 1.72E-12 | 2.40E-21 | 0.001232707 | 0.009218804 |
| **PTX3** | 10.82880924 | 1.798834624 | 65.18837699 | 0.009294247 |
| **HPRT1** | 13.24306202 | 1.889419135 | 92.82148593 | 0.00931144 |
| **CASP3** | 19.21606112 | 2.063281602 | 178.9658787 | 0.009427724 |
| **LGALS8** | 10.69804358 | 1.767415734 | 64.75450806 | 0.009882662 |
| **PTMA** | 1.235567744 | 1.051997532 | 1.451170372 | 0.009946829 |

**Table S2.** The 11 risk ISGs with the prognostic values in OSCC, ranked in the ascending order of p value.

| **Gene** | **coef** | **HR** | **HR.95L** | **HR.95H** | **P value** |
| --- | --- | --- | --- | --- | --- |
| **CXCL8** | 0.288485 | 1.334404905 | 1.15758651 | 1.538232 | 6.96E-05 |
| **TLR3** | 12.98194 | 434495.7834 | 235.284244 | 8.02E+08 | 0.000717 |
| **IL22** | 111.5162 | 2.70E+48 | 2.1766E+16 | 3.34E+80 | 0.003099 |
| **ORMDL3** | 2.390233 | 10.91604006 | 2.06792233 | 57.62302 | 0.004864 |
| **FGFR3** | 0.461974 | 1.58720361 | 1.13669769 | 2.216258 | 0.006684 |
| **CTLA4** | -34.1002 | 1.55E-15 | 1.35E-26 | 0.000178 | 0.008682 |
| **HPRT1** | 2.880587 | 17.8247369 | 1.8793766 | 169.0567 | 0.012085 |
| **BGLAP** | -313.988 | 4.33E-137 | 6.88E-246 | 2.73E-28 | 0.014029 |
| **CALCA** | 15.86313 | 7749419.427 | 4.07851285 | 1.47E+13 | 0.031513 |
| **SPHK1** | -1.54105 | 0.21415711 | 0.04455009 | 1.029477 | 0.054392 |
| **INHBB** | 3.34849 | 28.45972678 | 0.92025887 | 880.1394 | 0.055812 |

**Table S3.** The C-index values and time-dependent AUC values of 11 prognosis-related ISGs shown in the ROC curves.

| **gene** | **AUC_3year** | **AUC_5year** | **AUC_10year** | **C_index** |
| --- | --- | --- | --- | --- |
| **BGLAP** | 0.585908399 | 0.527998596 | 0.612412293 | 0.592171831 |
| **CALCA** | 0.58733954 | 0.518317073 | 0.499597736 | 0.545171199 |
| **CTLA4** | 0.570636586 | 0.507994334 | 0.629072855 | 0.567192158 |
| **CXCL8** | 0.557217335 | 0.46340983 | 0.426444354 | 0.534759238 |
| **FGFR3** | 0.58820389 | 0.645088423 | 0.619108408 | 0.566650104 |
| **HPRT1** | 0.644589888 | 0.679889265 | 0.617186862 | 0.544516216 |
| **IL22** | 0.495342866 | 0.514540836 | 0.450345297 | 0.502032704 |
| **INHBB** | 0.572947112 | 0.520038234 | 0.610548316 | 0.513054476 |
| **ORMDL3** | 0.62054251 | 0.680229337 | 0.710076278 | 0.544696901 |
| **SPHK1** | 0.556439107 | 0.575223459 | 0.623005443 | 0.571483422 |
| **TLR3** | 0.560633014 | 0.61855145 | 0.648047511 | 0.575887614 |

**Table S4. The clinical characteristics of OSCC samples (n=317) from TCGA database.**

| **Characteristics** | **n** | **%** |
| --- | --- | --- |
| **Age** |  |  |
| 19-49 | 51 | 9.9% |
| 50-59 | 84 | 16.2% |
| 60-69 | 95 | 18.4% |
| 70-90 | 86 | 16.6% |
| Not Available | 1 | 0.2% |
| **Gender** |  |  |
| Male | 211 | 66.6% |
| Female | 106 | 33.4% |
| **Pathologic_stage** |  |  |
| Stage I | 19 | 6.0% |
| Stage II | 54 | 17.0% |
| Stage III | 57 | 18.0% |
| Stage IV | 164 | 51.7% |
| Discrepancy | 1 | 0.3% |
| Not Available | 22 | 6.9% |
| **Clinical_stage** |  |  |
| Stage I | 12 | 3.8% |
| Stage II | 77 | 24.3% |
| Stage III | 63 | 19.9% |
| Stage IV | 157 | 49.5% |
| Not Available | 8 | 2.5% |
| **Grade** |  |  |
| G1 | 50 | 15.8% |
| G2 | 198 | 62.5% |
| G3 | 65 | 20.5% |
| GX | 3 | 0.9% |
| Not Available | 1 | 0.3% |
| **Vital status** |  |  |
| Alive | 203 | 64.0% |
| Death | 114 | 36.0% |

**Table S5. The clinical characteristics of OSCC samples (n=40) from ICGC database.**

| **Characteristics** | **n** | **%** |
| --- | --- | --- |
| **Age** |  |  |
| 19-49 | 21 | 52.5% |
| 50-59 | 11 | 27.5% |
| 60-69 | 7 | 17.5% |
| 70-90 | 1 | 2.5% |
| **Gender** |  |  |
| Male | 34 | 85.0% |
| Female | 6 | 15.0% |
| **Stage** |  |  |
| Stage I |  |  |
| Stage II |  |  |
| Stage III |  |  |
| Stage IV |  |  |
| **Vital status** |  |  |
| Alive | 32 | 80.0% |
| Death | 8 | 0.0% |

**Table S6. The clinical characteristics of OSCC samples (n=97) from** **GSE41613 dataset.**

| **Characteristics** | **n** | **%** |
| --- | --- | --- |
| **Age** |  |  |
| 19-49 | 22 | 22.7% |
| 50-59 | 28 | 28.9% |
| 60-69 | 27 | 27.8% |
| 70-88 | 20 | 20.6% |
| **Gender** |  |  |
| Male | 66 | 68.0% |
| Female | 31 | 32.0% |
| **Stage** |  |  |
| I/II | 41 | 30.9% |
| III/IV | 56 | 15.5% |
| **Vital status** |  |  |
| Alive | 46 | 47.4% |
| OSCC death | 30 | 30.9% |
| Death: other causes | 14 | 14.4% |
| Death: unknown cause | 7 | 7.2% |

**Table S7. The clinical characteristics of OSCC samples (n=71) from GSE42743 dataset.**

| **Characteristics** | **n** | **%** |
| --- | --- | --- |
| **Age** |  |  |
| 19-49 | 20 | 28.2% |
| 50-59 | 15 | 21.1% |
| 60-69 | 19 | 26.8% |
| 70-90 | 17 | 23.9% |
| **Gender** |  |  |
| Male | 55 | 77.5% |
| Female | 16 | 22.5% |
| **Stage** |  |  |
| Stage I | 3 | 4.2% |
| Stage II | 16 | 22.5% |
| Stage III | 15 | 21.1% |
| Stage IV | 37 | 52.1% |
| **Vital status** |  |  |
| Alive | 31 | 43.7% |
| OSCC death | 22 | 31.0% |
| Death: other causes | 10 | 14.1% |
| Death: unknown cause | 8 | 11.3% |

**Table S8. The clinical characteristics of OSCC samples (n=14) from GSE75538 dataset.**

| **Characteristics** | **n** | **%** |
| --- | --- | --- |
| **Age** |  |  |
| 19-49 | 10 | 71.4% |
| 50-59 | 1 | 7.1% |
| 60-69 | 1 | 7.1% |
| 70-90 | 2 | 14.3% |
| **Gender** |  |  |
| Male | 8 | 57.1% |
| Female | 6 | 42.9% |
| **Vital status** |  |  |
| Alive | 11 | 78.6% |
| Death | 3 | 21.4% |

**Table S9.** The parameter settings of the SVM algorithm.

|  | **Methods (Click to enter the official webpage)** | **Methods described** | **The parameters we used** | **Parameters to describe** |
| --- | --- | --- | --- | --- |
| 1 | [train_test_split](https://scikit-learn.org/stable/modules/generated/sklearn.model_selection.train_test_split.html?highlight=train_test_split#sklearn.model_selection.train_test_split) | Split arrays or matrices into random train and test subsets | *arrays | Allowed inputs are lists, numpy arrays, scipy-sparse matrices or pandas dataframes.  In *array, the * symbol allows the parameter array to accept multiple data sets.  Example: train_test_split (data,data2) train_test_split(data,data2,data3) |
|  |  |  | test_size | f float, should be between 0.0 and 1.0 and represent the proportion of the dataset to include in the test split. If int, represents the absolute number of test samples. If None, the value is set to the complement of the train size. If train_size is also None, it will be set to 0.25. |
|  |  |  | random_state | Controls the shuffling applied to the data before applying the split. Pass an int for reproducible output across multiple function calls. |
| 2 | [svm.SVC](https://scikit-learn.org/stable/modules/generated/sklearn.svm.SVC.html?highlight=svc#sklearn.svm.SVC) | The sklearn.svm module includes Support Vector Machine algorithms. C-Support Vector Classification. | C | Regularization parameter. The strength of the regularization is inversely proportional to C. Must be strictly positive. The penalty is a squared l2 penalty. |
|  |  |  | kernel | pecifies the kernel type to be used in the algorithm. It must be one of ‘linear’, ‘poly’, ‘rbf’, ‘sigmoid’, ‘precomputed’ or a callable. If none is given, ‘rbf’ will be used. If a callable is given it is used to pre-compute the kernel matrix from data matrices; that matrix should be an array of shape (n_samples, n_samples). |
|  |  |  | gamma | Kernel coefficient for ‘rbf’, ‘poly’ and ‘sigmoid’. |
|  |  |  | decision_function_shape | Whether to return a one-vs-rest (‘ovr’) decision function of shape (n_samples, n_classes) as all other classifiers, or the original one-vs-one (‘ovo’) decision function of libsvm which has shape (n_samples, n_classes * (n_classes - 1) / 2). However, one-vs-one (‘ovo’) is always used as multi-class strategy. The parameter is ignored for binary classification. |
|  |  |  | random_state | int, RandomState instance or None, default=None |
| 3 | [GridSearchCV](https://scikit-learn.org/stable/modules/generated/sklearn.model_selection.GridSearchCV.html?highlight=gridsearchcv#sklearn.model_selection.GridSearchCV) | Exhaustive search over specified parameter values for an estimator. | estimator | This is assumed to implement the scikit-learn estimator interface. Either estimator needs to provide a score function, or scoring must be passed. |
|  |  |  | param_grid | Dictionary with parameters names (str) as keys and lists of parameter settings to try as values, or a list of such dictionaries, in which case the grids spanned by each dictionary in the list are explored. This enables searching over any sequence of parameter settings. |
|  |  |  | cv | Determines the cross-validation splitting strategy |
|  |  |  | n_jobs | Number of jobs to run in parallel. None means 1 unless in a joblib.parallel_backend context. -1 means using all processors |
|  |  |  | scoring | Strategy to evaluate the performance of the cross-validated model on the test set |
|  |  |  | verbose | Controls the verbosity: the higher, the more messages |
| 4 | [fit](https://scikit-learn.org/stable/modules/generated/sklearn.svm.SVC.html?highlight=fit#sklearn.svm.SVC.fit) | Fit the SVM model according to the given training data. | X | Training vectors, where n_samples is the number of samples and n_features is the number of features. For kernel=”precomputed”, the expected shape of X is (n_samples, n_samples). |
|  |  |  | y | Target values (class labels in classification, real numbers in regression). |
| 5 | [predict](https://scikit-learn.org/stable/modules/generated/sklearn.svm.SVC.html?highlight=predict#sklearn.svm.SVC.predict) | erform classification on samples in X. For an one-class model, +1 or -1 is returned. | X | For kernel=”precomputed”, the expected shape of X is (n_samples_test, n_samples_train). |
| 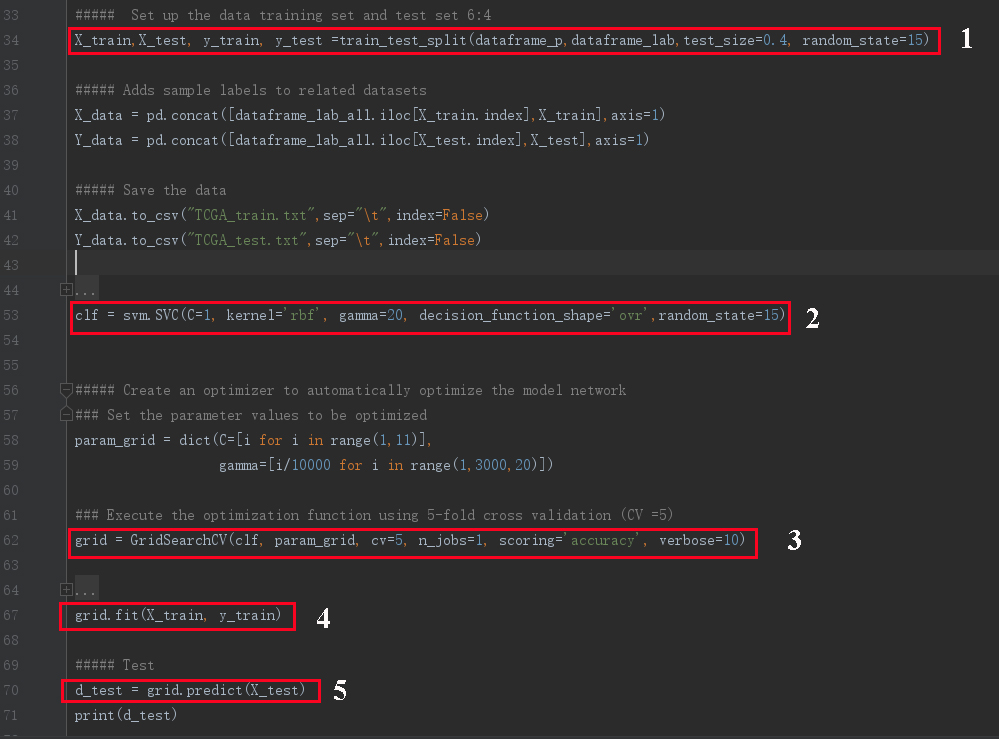 | | | | |

**Table S10.** The parameter settings of the autoencoder algorithm.

|  | **Methods** (Click to enter the official documentation) | **Methods described** | **The parameters we used** | **Parameters to describe** |
| --- | --- | --- | --- | --- |
| 1 | [Input](https://keras.io/api/layers/core_layers/input/) | Input is used to instantiate a Keras tensor. | shape | A shape tuple (integers), not including the batch size. For instance, shape=(32,) indicates that the expected input will be batches of 32-dimensional vectors. Elements of this tuple can be None; 'None' elements represent dimensions where the shape is not known. |
| 2 | [Dense](https://keras.io/api/layers/core_layers/dense/) | Create Just your regular dense -connected layer. | units | Positive integer, dimensionality of the output space. |
|  |  |  | activation | Activation function to use. If you don't specify anything, no activation is applied (ie. "linear" activation: a(x) = x). |
|  |  |  | activity_regularizer | Regularizer function applied to the output of the layer (its "activation"). |
| 3 | [regularizers.l1_l2](https://keras.io/api/layers/regularizers/#l1_l2-function) | Create a regularizer that applies both L1 and L2 penalties. | l1 | The L1 regularization penalty is computed as: loss = l1 * reduce_sum(abs(x)) |
|  |  |  | l2 | The L2 regularization penalty is computed as: loss = l2 * reduce_sum(square(x)) |
| 4 | [Dropout](https://tensorflow.google.cn/swift/api_docs/Structs/Dropout?hl=en) | A dropout layer.  Dropout consists in randomly setting a fraction of input units to 0 at each update during training time, which helps prevent overfitting. | rate | Floating between 0 and 1.Proportion of input that needs to be discarded |
| 5 | [Model](https://keras.io/api/models/model/#model-class) | Model groups layers into an object with training and inference features. | inputs | The input(s) of the model: a keras.Input object or list of keras.Input objects. |
|  |  |  | outputs | The output(s) of the model. See Functional API example below. |
| 6 | [Model.compile](https://keras.io/api/models/model_training_apis/#compile-method) | Configures the model for training. Model.compile The Model refers to the name of the Model object that you create | optimizer | String (name of optimizer) or optimizer instance. See tf.keras.optimizers |
|  |  |  | loss | String (name of objective function), objective function or tf.keras.losses.Loss instance. See tf.keras.losses. |
|  |  |  | metrics | List of metrics to be evaluated by the model during training and testing. Each of this can be a string (name of a built-in function), function or a tf.keras.metrics.Metric instance. |
| 7 | [Model.fit](https://keras.io/api/models/model_training_apis/#fit-method) | Trains the model for a fixed number of epochs (iterations on a dataset). Model.compile The Model refers to the name of the Model object that you create | x | x: Input data. It could be: ·A Numpy array (or array-like), or a list of arrays (in case the model has multiple inputs). ·A TensorFlow tensor, or a list of tensors (in case the model has multiple inputs). ·A dict mapping input names to the corresponding array/tensors, if the model has named inputs. ·A tf.data dataset. Should return a tuple of either (inputs, targets) or (inputs, targets, sample_weights). ·A generator or keras.utils.Sequence returning (inputs, targets) or (inputs, targets, sample_weights). A more detailed description of unpacking behavior for iterator types (Dataset, generator, Sequence) is given below. |
|  |  |  | y | Target data. Like the input data x, it could be either Numpy array(s) or TensorFlow tensor(s). It should be consistent with x (you cannot have Numpy inputs and tensor targets, or inversely). If x is a dataset, generator, or keras.utils.Sequence instance, y should not be specified (since targets will be obtained from x). |
|  |  |  | epochs | Integer. Number of epochs to train the model |
| 8 | [Model.predict](https://keras.io/api/models/model_training_apis/#predict-method) | Generates output predictions for the input samples. Model.compile The Model refers to the name of the Model object that you create | x | Input samples. It could be: ·A Numpy array (or array-like), or a list of arrays (in case the model has multiple inputs). ·A TensorFlow tensor, or a list of tensors (in case the model has multiple inputs). ·A tf.data dataset. ·A generator or keras.utils.Sequence instance. A more detailed description of unpacking behavior for iterator types (Dataset, generator, Sequence) is given in the Unpacking behavior for iterator-like inputs section of Model.fit |
| 9 | [Model.evaluate](https://keras.io/api/models/model_training_apis/#evaluate-method) | Returns the loss value & metrics values for the model in test mode Model.compile The Model refers to the name of the Model object that you create | x | Similar to the data structure and functionality required by 7. Model.fit(x,y) |
|  |  |  | y | Similar to the data structure and functionality required by 7. Model.fit(x,y) |
| 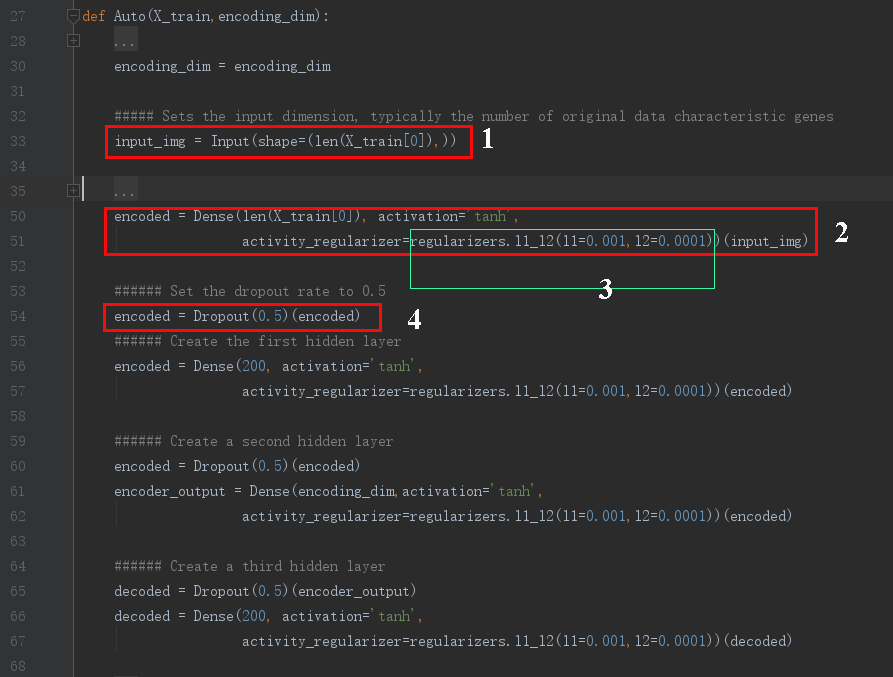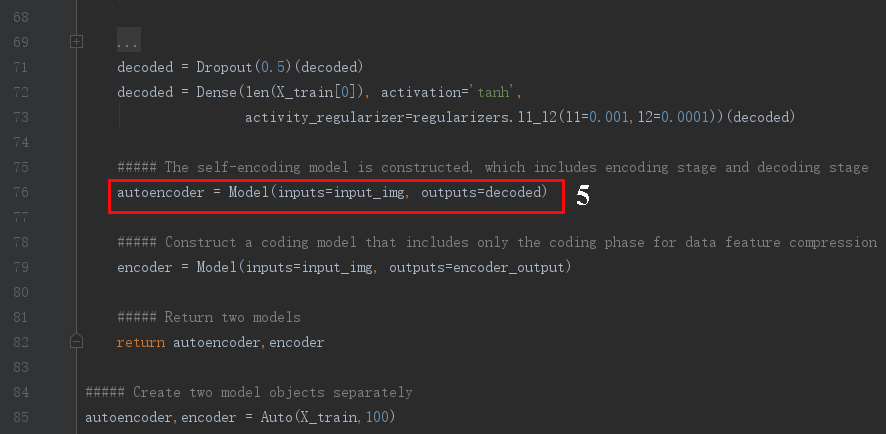 | | | | |
| 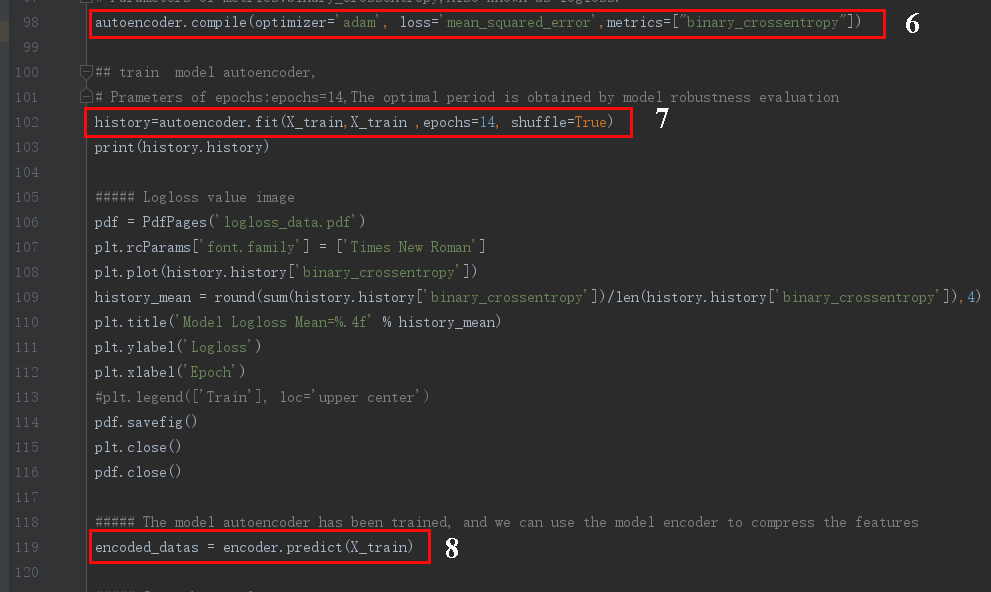 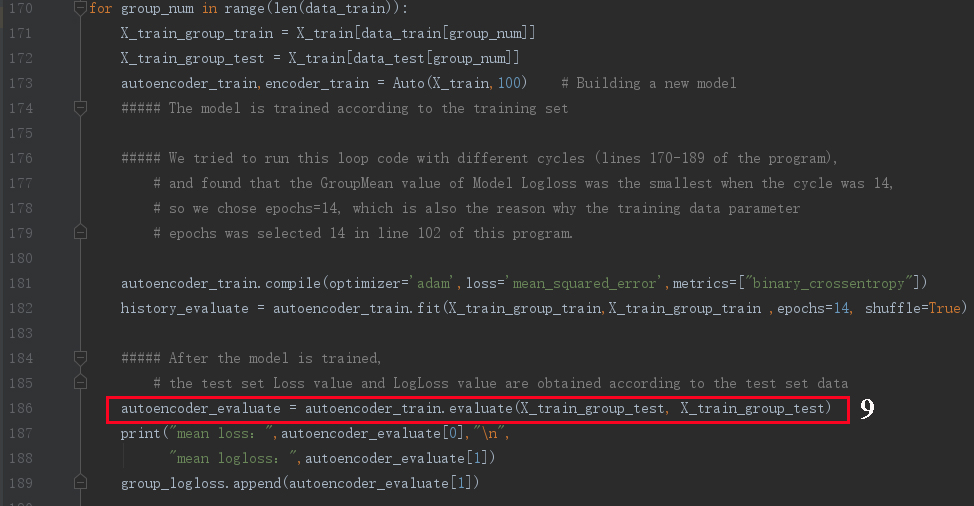 | | | | |

**Table S11.** The performance of the SVM classifier on training and test set in TCGA cohort, as well as the four external confirmation cohorts.

| **data** | **Samples** | **c.index** | **c.index_lower** | **c.index_upper** | **Brier_score** | **Log.rank_pvalue** |
| --- | --- | --- | --- | --- | --- | --- |
| TCGA_Training | 197 | 0.807714204 | 0.763007986 | 0.852420423 | 0.128467367 | 4.15E-20 |
| TCGA_Test | 133 | 0.699353081 | 0.622371827 | 0.776334336 | 0.14112538 | 0.000173202 |
| TCGA_All | 330 | 0.690532266 | 0.643654516 | 0.737410015 | 0.14097987 | 1.94E-12 |
| GSE41613 | 97 | 0.855626327 | 0.799205322 | 0.912047331 | 0.095814561 | 3.62E-08 |
| GSE42743 | 71 | 0.86637931 | 0.802497462 | 0.930261158 | 0.071131701 | 7.55E-08 |
| GSE75538 | 14 | 0.483516484 | 0.339264264 | 0.627768703 | 0.187255156 | 0.729088663 |
| ICGC | 40 | 0.387179487 | 0.280583347 | 0.493775628 | 0.092957991 | 0.088125051 |

**Table S12.** The model performance comparison between Deep learning-based model with the PCA-based model.

| **Data** | **Method** | **Samples** | **c.index** | **c.index_lower** | **c.index_upper** | **Brier_score** | **Log.rank_pvalue** |
| --- | --- | --- | --- | --- | --- | --- | --- |
| TCGA | DL | 330 | 0.769665568 | 0.725005962 | 0.814325173 | 0.135906584 | 4.91E-22 |
| TCGA | PCA | 330 | 0.688440974 | 0.643747053 | 0.733134894 | 0.111433297 | 5.36E-18 |

**Table S13.** The all DEGs dysregulated betwween the two subytpes of OSCC samples, ranked by the ascending order of p value.

| **ISGs-DEGs** | **foldChange** | **log2FoldChange** | **pval** | **padj** |
| --- | --- | --- | --- | --- |
| **CST2** | 509.0180384 | 8.991572973 | 1.06E-161 | 1.25E-158 |
| **LCN1** | 621.7810482 | 9.280262834 | 6.74E-158 | 3.98E-155 |
| **MUC6** | 137.4838422 | 7.103118266 | 7.35E-56 | 2.89E-53 |
| **PIP** | 14.53135248 | 3.861097081 | 1.56E-28 | 4.61E-26 |
| **ACTA1** | 8.298119418 | 3.052784419 | 4.16E-24 | 9.81E-22 |
| **FN1** | 3.385102048 | 1.759199326 | 2.84E-23 | 5.59E-21 |
| **HSD3B1** | 75.49629552 | 6.23833395 | 3.07E-22 | 5.17E-20 |
| **S100A1** | 6.278807785 | 2.650490647 | 6.35E-21 | 9.37E-19 |
| **PDK4** | 7.038390391 | 2.815245537 | 9.82E-21 | 1.29E-18 |
| **TTN** | 7.554392714 | 2.917315784 | 2.08E-20 | 2.45E-18 |
| **CSN3** | 21.58132261 | 4.431711378 | 1.34E-18 | 1.44E-16 |
| **COMP** | 3.491101678 | 1.803682376 | 1.95E-16 | 1.92E-14 |
| **LTF** | 4.570567166 | 2.192373202 | 3.85E-16 | 3.50E-14 |
| **RYR1** | 5.043898284 | 2.334539182 | 7.27E-14 | 6.13E-12 |
| **AMOT** | 4.195800194 | 2.068945978 | 4.80E-12 | 3.77E-10 |
| **NCAM1** | 3.194217112 | 1.675462377 | 7.95E-12 | 5.87E-10 |
| **CHRNA1** | 3.682159513 | 1.880552126 | 9.46E-12 | 6.57E-10 |
| **LGALS14** | 53.12674016 | 5.731366287 | 9.94E-11 | 6.51E-09 |
| **PLA2G2A** | 3.751092963 | 1.907311018 | 1.48E-10 | 9.19E-09 |
| **CALCA** | 14.15246279 | 3.822981225 | 7.54E-10 | 4.45E-08 |
| **RARRES1** | 2.927958868 | 1.549895287 | 1.26E-08 | 7.07E-07 |
| **TNFAIP6** | 2.517162416 | 1.331798307 | 1.56E-08 | 8.05E-07 |
| **SPARC** | 2.17736791 | 1.1225852 | 1.57E-08 | 8.05E-07 |
| **MUC16** | 0.429251932 | -1.220103466 | 1.83E-08 | 8.98E-07 |
| **RAMP1** | 2.937550776 | 1.554613789 | 3.45E-08 | 1.63E-06 |
| **CXCL12** | 2.592531508 | 1.374361523 | 8.32E-08 | 3.78E-06 |
| **GSTA1** | 2.814499132 | 1.492878203 | 1.09E-07 | 4.76E-06 |
| **MAP1A** | 2.304088155 | 1.204195916 | 2.07E-07 | 8.72E-06 |
| **MAPT** | 3.619438907 | 1.855766065 | 2.73E-07 | 1.11E-05 |
| **AGT** | 3.937112864 | 1.97713807 | 7.67E-07 | 3.02E-05 |
| **CCDC80** | 2.393191227 | 1.258935679 | 1.56E-06 | 5.95E-05 |
| **CHI3L1** | 2.270203176 | 1.18282142 | 2.44E-06 | 9.01E-05 |
| **PDE4B** | 2.212603538 | 1.145744967 | 3.32E-06 | 0.000118737 |
| **IL6** | 2.139387592 | 1.097197879 | 6.54E-06 | 0.000226879 |
| **NOS2** | 0.196515725 | -2.347283331 | 1.20E-05 | 0.000403528 |
| **ZEB1** | 2.110151062 | 1.077346283 | 1.48E-05 | 0.000486401 |
| **PPARGC1A** | 3.495369633 | 1.805445028 | 1.79E-05 | 0.000564753 |
| **MMP2** | 2.098568606 | 1.069405629 | 1.82E-05 | 0.000564753 |
| **FAM107A** | 3.14385975 | 1.652536859 | 2.02E-05 | 0.000610274 |
| **THY1** | 2.093146068 | 1.065672992 | 3.59E-05 | 0.001034577 |
| **SCN5A** | 3.574613235 | 1.837787154 | 4.90E-05 | 0.001345972 |
| **CRYAB** | 2.53190246 | 1.340221827 | 5.06E-05 | 0.001357409 |
| **TF** | 2.3898822 | 1.256939508 | 5.85E-05 | 0.001535077 |
| **DCN** | 2.108213531 | 1.076020998 | 7.01E-05 | 0.001798714 |
| **CLU** | 2.401622887 | 1.264009631 | 8.23E-05 | 0.002065934 |
| **NDRG2** | 2.738519701 | 1.453396259 | 9.00E-05 | 0.00221321 |
| **G0S2** | 2.067297866 | 1.047746274 | 0.000121528 | 0.002811828 |
| **FGF7** | 2.725134855 | 1.446327624 | 0.000162738 | 0.00369289 |
| **LBP** | 4.952874099 | 2.308265948 | 0.000214292 | 0.004597539 |
| **IDO1** | 0.489510397 | -1.030588593 | 0.000313571 | 0.006491479 |
| **DPP4** | 2.106542814 | 1.074877238 | 0.00035681 | 0.007259232 |
| **IL22** | 0.122611171 | -3.027837663 | 0.000414882 | 0.008297643 |
| **CSN2** | 7.20085162 | 2.848167539 | 0.000424123 | 0.008341085 |
| **TNS1** | 2.024603529 | 1.017639418 | 0.000432711 | 0.008370481 |
| **PGR** | 3.496365664 | 1.805856075 | 0.000629541 | 0.0114814 |
| **MITF** | 2.482731076 | 1.311928 | 0.00063245 | 0.0114814 |
| **ALB** | 2.846937872 | 1.50941101 | 0.000672216 | 0.012018402 |
| **KIT** | 2.046364442 | 1.033063101 | 0.000809163 | 0.013837865 |
| **KCNJ11** | 3.40239835 | 1.76655206 | 0.000998056 | 0.016587405 |
| **NOX4** | 2.077211867 | 1.054648372 | 0.002227688 | 0.034138602 |
| **S100A4** | 2.02825804 | 1.020241207 | 0.002601949 | 0.038378742 |
| **EGF** | 2.963427588 | 1.567266805 | 0.002846338 | 0.040959499 |
| **FEZF2** | 0.206785038 | -2.273796292 | 0.003620065 | 0.04909973 |

**Table S14.** The topological characteristics of the nodes in the ISGs-DEGs related PPI network. The listed nodes in this table include the top 20 ISGs-upregulated DEGs, top 20 ISGs-downregulated DEGs, and top 20 ISGs-non DEGs.

| **The topological characteristics of the top 20 ISGs-upregulated DEGs** | | | | | | |
| --- | --- | --- | --- | --- | --- | --- |
| **name** | **Degree** | **AverageShortestPathLength** | **BetweennessCentrality** | **ClosenessCentrality** | **ClusteringCoefficient** | **TopologicalCoefficient** |
| FN1 | 818 | 2.40014648 | 0.02909324 | 0.41664124 | 0.00917244 | 0.00528033 |
| ALB | 210 | 2.80541992 | 0.00966531 | 0.35645288 | 0.00719982 | 0.00994038 |
| ACTA1 | 190 | 2.54882813 | 0.00730824 | 0.39233716 | 0.0235589 | 0.01247946 |
| TTN | 136 | 2.60986328 | 0.00383797 | 0.38316183 | 0.02864924 | 0.01593605 |
| MAPT | 125 | 2.6233724 | 0.00232915 | 0.38118873 | 0.05870968 | 0.02035889 |
| CLU | 116 | 2.71704102 | 0.00420158 | 0.36804744 | 0.02608696 | 0.01691286 |
| AMOT | 108 | 2.74820964 | 0.0023905 | 0.36387326 | 0.01436483 | 0.0162841 |
| KIT | 102 | 2.78914388 | 6.03E-04 | 0.35853296 | 0.10211609 | 0.02539427 |
| CRYAB | 100 | 2.76953125 | 0.00415566 | 0.36107193 | 0.02343434 | 0.01792092 |
| PGR | 75 | 2.71582031 | 2.78E-04 | 0.36821287 | 0.10018018 | 0.02890846 |
| PPARGC1A | 73 | 2.69580078 | 9.25E-04 | 0.37094729 | 0.10464231 | 0.02980711 |
| S100A4 | 70 | 2.73925781 | 9.39E-04 | 0.36506239 | 0.03354037 | 0.02488089 |
| TF | 63 | 2.81884766 | 0.00211282 | 0.35475489 | 0.04147465 | 0.02445178 |
| MMP2 | 46 | 2.95092773 | 0.00134998 | 0.33887648 | 0.03671498 | 0.02809793 |
| DCN | 37 | 2.96191406 | 7.20E-04 | 0.33761952 | 0.05255255 | 0.03530844 |
| MAP1A | 37 | 2.70157878 | 5.90E-04 | 0.37015393 | 0.07957958 | 0.04458531 |
| MITF | 34 | 2.8655599 | 3.28E-04 | 0.34897194 | 0.0802139 | 0.04930663 |
| DPP4 | 32 | 3.22167969 | 9.06E-04 | 0.31039709 | 0.03629032 | 0.03967391 |
| NCAM1 | 32 | 2.7421875 | 0.0011778 | 0.36467236 | 0.06048387 | 0.04452207 |
| FAM107A | 31 | 3.13273112 | 2.79E-04 | 0.31921029 | 0.00430108 | 0.04404936 |
| **The topological characteristics of the top 20 ISGs-downregulated DEGs** | | | | | | |
| **name** | **Degree** | **AverageShortestPathLength** | **BetweennessCentrality** | **ClosenessCentrality** | **ClusteringCoefficient** | **TopologicalCoefficient** |
| NOS2 | 160 | 2.78092448 | 0.00177445 | 0.35959265 | 0.01823899 | 0.01836409 |
| MUC16 | 7 | 2.99031576 | 1.68E-04 | 0.33441285 | 0.19047619 | 0.16069364 |
| IDO1 | 6 | 3.00130208 | 1.75E-04 | 0.33318872 | 0 | 0.1984127 |
| IL22 | 4 | 4.06274414 | 1.65E-04 | 0.24613905 | 0 | 0.27678571 |
| FEZF2 | 1 | 3.34008789 | 0 | 0.29939332 | 0 | 0 |
| **The topological characteristics of the top 20 ISGs-non DEGs** | | | | | | |
| **name** | **Degree** | **AverageShortestPathLength** | **BetweennessCentrality** | **ClosenessCentrality** | **ClusteringCoefficient** | **TopologicalCoefficient** |
| NTRK1 | 1968 | 2.19946289 | 0.11243976 | 0.45465645 | 0.0037814 | 0.00301205 |
| JUN | 1504 | 2.24503581 | 0.06732348 | 0.44542719 | 0.00711255 | 0.00396764 |
| TP53 | 1079 | 2.23763021 | 0.0565704 | 0.44690137 | 0.01229064 | 0.00487542 |
| MYC | 1027 | 2.29475911 | 0.05462615 | 0.43577559 | 0.00859066 | 0.00455508 |
| EGFR | 1009 | 2.27124023 | 0.04829181 | 0.44028808 | 0.01068361 | 0.00480828 |
| HSP90AA1 | 856 | 2.24243164 | 0.03573635 | 0.44594447 | 0.01774608 | 0.00593926 |
| ESR1 | 824 | 2.29378255 | 0.02746309 | 0.43596112 | 0.01919629 | 0.00625332 |
| SUMO1 | 802 | 2.3819987 | 0.03177737 | 0.41981551 | 0.01202674 | 0.00564109 |
| CDK2 | 746 | 2.37198893 | 0.02168111 | 0.42158713 | 0.01224967 | 0.00582918 |
| HSP90AB1 | 738 | 2.2972819 | 0.02612199 | 0.43529703 | 0.01809872 | 0.00640661 |
| VCAM1 | 673 | 2.5789388 | 0.01082952 | 0.38775639 | 0.00820332 | 0.00693509 |
| HDAC1 | 640 | 2.34016927 | 0.02539737 | 0.42731952 | 0.01983079 | 0.00632034 |
| TRAF6 | 550 | 2.41577148 | 0.01921156 | 0.41394644 | 0.0148104 | 0.00663194 |
| ITGA4 | 528 | 2.62410482 | 0.00804132 | 0.38108234 | 0.01270053 | 0.00789362 |
| EP300 | 525 | 2.36409505 | 0.01756326 | 0.42299484 | 0.0280189 | 0.00784637 |
| ESR2 | 504 | 2.62646484 | 0.0140041 | 0.38073991 | 0.00729748 | 0.00681447 |
| SRC | 488 | 2.36499023 | 0.01662154 | 0.42283473 | 0.02672771 | 0.00777527 |
| KRAS | 461 | 2.65332031 | 0.02802588 | 0.37688627 | 0.00278223 | 0.00546921 |
| ILK | 456 | 2.58186849 | 0.01102325 | 0.3873164 | 0.01240602 | 0.00822231 |
| HSPA8 | 451 | 2.30021159 | 0.01573448 | 0.43474261 | 0.03681695 | 0.00963164 |
